# Identification and quantification of irreversibility in stochastic systems

**DOI:** 10.1039/d5cp04712a

**Published:** 2026-04-08

**Authors:** Aishani Ghosal, Gili Bisker

**Affiliations:** a School of Chemical Sciences, National Institute of Science Education and Research, Khurdha, Jatni Rd Bhubaneswar 752050 India aishanig@niser.ac.in; b Department of Physics, Washington University in St. Louis 1 Brookings Drive St. Louis MO USA; c School of Biomedical Engineering, Faculty of Engineering, Tel Aviv University Tel Aviv 69978 Israel bisker@tauex.tau.ac.il; d Center for Physics and Chemistry of Living Systems, Tel Aviv University Tel Aviv 6997801 Israel; e Center for Nanoscience and Nanotechnology, Tel Aviv University Tel Aviv 6997801 Israel; f Center for Light-Matter Interaction, Tel Aviv University Tel Aviv 6997801 Israel; g Center for Computational Molecular and Materials Science, Tel Aviv University Tel Aviv 6997801 Israel; h Sagol School for Neuroscience, Tel Aviv University Tel Aviv 6997801 Israel

## Abstract

Advances in single-molecule measurements, active-matter control, and nonequilibrium statistical mechanics are transforming our understanding of thermodynamics in small, strongly fluctuating systems. Biological molecular motors, driven chemical-reaction networks (*e.g.*, gene regulation), artificial active matter, autonomous engines, and synthetic nanomachines all operate *via* inherently irreversible, dissipative processes in noisy environments, while producing entropy. Quantifying this entropy production (EP) has therefore become central to understanding both the physical limits and design principles of nanoscale systems. This review surveys principled routes to characterize and quantify EP from time-series and trajectory data. Because experimental observables are often coarse-grained and only partially resolve the underlying dynamics, we discuss how dissipation can be inferred from incomplete information, and how coarse-graining systematically biases EP estimates. This overview maps the current toolkit for estimating EP and outlines open challenges in unifying inference approaches to obtain reliable and tight bounds on EP in living and engineered nanoscale systems.

## Abstract 2

This review summarizes methods for estimating entropy production rates from observed trajectories and coarse-grained time series.

## Graphical abstract

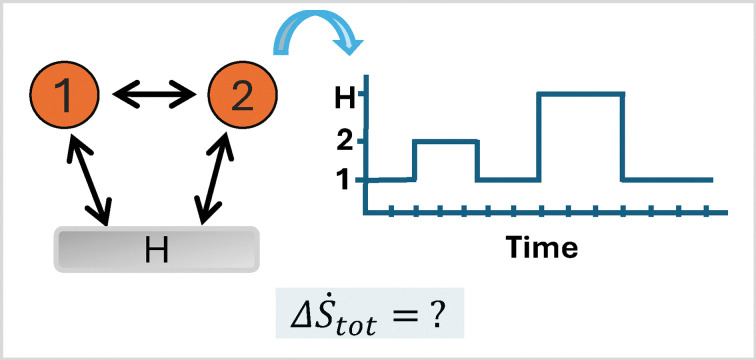

## 1 Introduction and overview

The concept of irreversibility, a cornerstone of modern thermodynamics, was not present in classical thermodynamics, which emerged in the 19th century through the foundational work of Carnot, Clausius, Joule, Helmholtz, Kelvin, and Gibbs. Classical thermodynamics primarily addressed equilibrium states and quasi-static processes, focusing on energy exchange without accounting for time-dependent or directional (arrow of time) processes. In contrast, the formal theory of irreversible processes was developed in the 20th century by Lars Onsager,^1,2^ Théophile De Donder,^3^ Ilya Prigogine,^4–6^ and others, laying the foundation for modern thermodynamics.^4,5,7^ This framework introduced linear irreversible thermodynamics to describe systems close to equilibrium and was later extended to capture the behavior of systems operating far from equilibrium.^8^

Modern thermodynamics provides a basis for understanding how highly ordered structures can arise and persist in nature—phenomena that classical frameworks could not explain. Irreversible processes, such as those involved in biological evolution, are fundamental to the emergence and maintenance of such structures. These processes can both generate^9^ and degrade order,^10^ with sustained order requiring continuous dissipation of energy. In this sense, irreversibility is intimately linked to the flow of energy and the arrow of time. To further generalize the thermodynamics of small-scale, highly fluctuating systems, the field of stochastic thermodynamics has emerged as a sub-discipline of nonequilibrium statistical physics at the end of the twentieth century.^11^ It extends the principles of thermodynamics, which were originally formulated for ensemble-averaged systems near equilibrium, to individual trajectories of systems operating far-from-equilibrium.^12,13^ Early developments focused on steady-state systems^14,15^ and ensemble averages,^16^ leading to the discovery of key results such as fluctuation theorems.^17–23^

A significant advancement in this field is the formulation of thermodynamics at the trajectory level. In the framework of stochastic energetics, introduced by Sekimoto,^11^ thermodynamic quantities such as work, heat, and entropy production are defined along single trajectories. Within this framework, even the first and second laws of thermodynamics have been recast in terms of path-dependent quantities.^24,25^ These theoretical predictions have been experimentally verified through advances in single-molecule manipulation and detection techniques.^26–30^ More recently, thermodynamic uncertainty relations (TUR)^31–34^ have emerged, offering fundamental bounds on the trade-offs between precision and dissipation in fluctuating systems. These bounds have been extended to a wide range of systems, including those with multiple time-integrated observables,^35^ underdamped dynamics,^36^ broken time reversal symmetry,^37^ and non-Markovian (memory-retaining) behavior.^38^ Additional relations, such as the dissipation-time uncertainty relation^39^ and energy-efficiency trade-offs,^40^ have proven particularly relevant for analyzing and constraining the performance of biological and artificial nanoscale systems.

The framework of stochastic thermodynamics has been successfully applied across a wide range of systems to quantify dissipation. In biological systems, dissipation has been investigated in contexts such as Darwinian dynamics,^41^ biochemical reaction networks,^42^ self-replication,^43,44^ active cytoskeleton material,^45^ population dynamics,^46^ ecological systems, sensory adaption,^47^ and even neural^48^ and brain function.^49^ Beyond biology, dissipation estimates have been crucial in understanding the thermodynamic costs of information processing,^47,50–54^ including contexts involving Shannon information,^55^ information geometry,^56,57^ and information ratchets.^58–61^ Studies have also extended to the thermodynamic cost of computation and cognition, including analyses of Turing machines.^62–65^ Additionally, stochastic thermodynamics has been applied to study dissipation in stochastic transport and optimal transport theory,^66,67^ uncovering profound connections between physical cost and the geometry of probability flows. Collectively, these applications highlight the versatility of stochastic thermodynamics in quantifying dissipation across a wide range of systems. Together, these diverse applications reveal the versatility of stochastic thermodynamics across biological, informational, and physical domains.

Both natural and synthetic nanoscale systems face the same fundamental energetic constraints: dissipation sets limits on precision and performance. This unifying perspective makes dissipation not just a cost, but a design principle. Small-scale living systems, such as biological molecular machines, function in highly fluctuating environments and rely on precise energy management to maintain robust performance. The thermodynamic cost of these processes—often quantified by dissipation—plays a critical role in determining how such systems can perform reliably under thermal noise,^68^ maintain a target nonequilibrium state,^69^ and other stochastic influences. Understanding dissipation is therefore essential for uncovering the design principles of molecular machines in biology and for guiding the development of efficient artificial Brownian motor^70^ or nanoscale devices. Recent advances in nanotechnology have intensified interest in dissipation, as researchers strive to engineer synthetic molecular machines capable of performing specific tasks, such as molecule synthesis, cargo transport, and vesicle fusion.^71^ These artificial systems, much like their biological counterparts, must operate under tight energetic constraints, making dissipation not only a measure of inefficiency but also a design constraint for functionality and control. As such, dissipation has become a key quantity in the design, optimization, and theoretical understanding of both natural and engineered molecular systems.

A significant challenge in analyzing small-scale systems lies in the limited spatiotemporal resolution of experimental techniques, particularly in single-molecule measurement.^72^ This limitation makes it difficult to access all relevant, driven degrees of freedom, resulting in only partial observations of the system's true dynamics. Consequently, the observed dynamics are inherently coarse-grained, representing a projection of the full microscopic evolution onto a lower-dimensional space.^73,74^

In molecular simulations, the term coarse-graining typically refers to a modeling strategy in which several atoms are grouped into a single effective particle. This reduction eliminates fine-scale degrees of freedom and interactions that are deemed irrelevant at the scale of interest. By smoothing the configurational landscape and reducing the dimensionality of the phase space, such coarse-grained models significantly accelerate molecular dynamics simulations of complex systems. The coarser the representation, the greater the computational gain. This form of coarse-graining is widely used to enable simulations over longer time and length scales. However, the notion of coarse-graining considered in this review does not arise from a deliberate modeling simplification, but from limited observational access to the underlying dynamics. Many nonequilibrium systems, particularly biological ones, operate across multiple timescales, and experimental techniques often cannot resolve all relevant microstates or transitions. As a result, certain degrees of freedom remain hidden, and the observed dynamics represent only a projection of the full microscopic process. Thus, coarse-graining reflects partial information due to finite spatiotemporal resolution or intrinsically unobservable states, rather than a reduction performed for computational efficiency.

In this context, coarse-graining reflects partial observational access to the system's dynamics: hidden degrees of freedom, unresolved transitions, or finite spatiotemporal resolution effectively project the full microscopic evolution onto a reduced description. Such observational coarse-graining introduces fundamental discrepancies between the observed dynamics and the underlying microscopic process, with direct consequences for the thermodynamic inference of irreversibility. In particular, dissipation estimated from coarse-grained data often underestimates the true entropy production, due to the loss of information about hidden or unobserved degrees of freedom.^75^ Understanding how coarse-graining affects dissipation has thus become a critical area of investigation in stochastic thermodynamics. Various methods have been developed to model coarse-grained systems, often differing in the quantities preserved during the coarse-graining procedure—such as total probability densities,^76^ fluxes,^74^ or transition rates.^77^ Recent efforts have focused on formulating coarse-graining frameworks that preserve thermodynamic consistency^78,79^ and enable reliable inference of dissipation from partial data.^80^ This has led to ongoing theoretical advancements aimed at extending stochastic thermodynamics to partially observed systems, bridging the gap between experimental accessibility and microscopic thermodynamic laws.

In this review, we discuss recent advances in the detection and quantification of irreversibility in small-scale systems, with a particular focus on the effects of coarse-graining on dissipation within the framework of stochastic thermodynamics. The review proceeds as follows: Section 2 outlines criteria for irreversibility; Section 3 examines dissipation estimation for fully resolved systems near equilibrium; Section 4 addresses the impact of coarse-graining and partial observations; Section 5 surveys theoretical and experimental advances in dissipation quantification; and Section 6 concludes.

## 2 Qualitative and quantitative criteria for nonequilibrium

Systems governed by time-independent conservative potentials relax to thermal equilibrium, characterized by a time-independent probability distribution and vanishing probability currents between all pairs of states, *i.e.*, detailed balance.^81^ Time-independent non-conservative driving breaks detailed balance and, in the long-time limit, leads to a non-equilibrium steady state (NESS) characterized by a stationary probability distribution with non-zero steady-state probability currents. Under explicitly time-dependent forces, however, the system generally does not reach a steady state, and the probability distribution remains time-dependent. In this section, we focus on criteria for identifying and characterizing NESS.

### 2.1 Broken detailed balance and time reversal symmetry

The principle of detailed balance (DB), which serves as a microscopic basis for thermodynamics, was identified by Ludwig Boltzmann.^82^ Systems at thermodynamic equilibrium are considered to follow DB, or the Kolmogorov criterion.^83^ DB refers to the reversible and pairwise balance of transition rates between any two discrete microstates, therefore, the ratio of products of the transition rates along the clockwise direction to counterclockwise direction equals unity. The latter results in a vanishing net transition flux or current. At NESS, in contrast, the probability distribution becomes time-independent, yet detailed balance is violated and persistent probability currents circulate through state space.

**Fig. 1:**
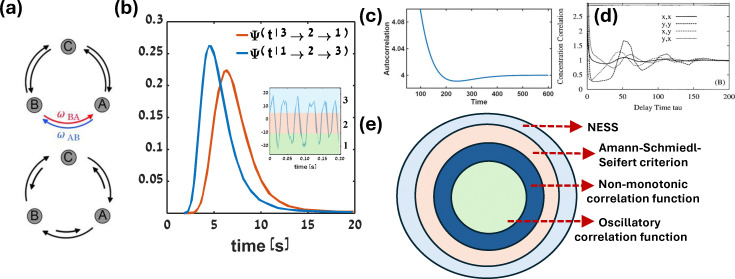
Criteria for irreversibility detection: (a) detailed balance with zero pairwise flux (top) and broken detailed balance with a non-zero net flux (bottom). This figure has been reproduced from ref. 85 with permission from the American Association for the Advancement of Science, copyright 2026. (b) An example of broken time-reversal symmetry, where a continuous variable is discretized into three states (as shown in the inset with different shaded colors, which are labeled by 1, 2, and 3) following a commonly used spatial coarse-graining of a positional degree of freedom *x*_1_, plotted as a function of time. The underlying process in the discretized space is modeled as a second-order semi-Markov process, and the asymmetry in the distribution of waiting times at state 2 for an upward transition (1 → 2 → 3) *versus* a downward transition (3 → 2 → 1) reflects the broken time-reversal symmetry. This figure has been reproduced from ref. 86 with permission from The Royal Society of Chemistry, copyright 2026. (c) A non-monotonic time autocorrelation function for a three-state kinetic cycle, indicative of a non-equilibrium steady state. This figure has been adapted from ref. 87 with permission from the American Physical Society, copyright 2026. (d) An oscillatory correlation function exhibited by an open chemical or biochemical reaction network. This figure has been reproduced from ref. 88 with permission from the National Academy of Sciences, copyright 2026. (e) A schematic view of the expansion of the parameter space defining a non-equilibrium steady state: the subset of system parameters for which the system exhibits an oscillatory correlation function lies within the parameter regime where the time autocorrelation function of system variables shows non-monotonic behavior. Furthermore, this regime is itself a subset of the parameter range identified by the Amann–Schmiedl–Seifert criteria or their generalizations. This figure has been adapted from ref. 89 with permission from the IOP Publishing Ltd copyright 2026.

If all microstates are experimentally accessible, distinguishing between equilibrium and NESS becomes trivial, provided sufficiently long trajectories are available. At equilibrium, the following DB equality holds at steady state:

1 *P*^ss^_*i*_*k*_*ji*_ =
*P*^ss^_*j*_*k*_*ij*_ ∀*i*,
*j* ≠ *i*

where the stationary probabilities at state *i* are denoted *P*^ss^_*i*_, which can be obtained from the fraction of time the system spends at a particular state, given a long trajectory, and the transition rate from state *i* to *j* is denoted as *k*_*ji*_. Under this condition, all pairwise probability currents vanish. By contrast, at NESS, detailed balance is broken and closed loops of probability flux persist.^84^ The stationary probability density is maintained by constant probability currents, reflecting sustained entropy production despite time-independent statistics. Fig. 1(a) illustrates the detailed balance criterion (top) and the broken detailed balance leading to net transition flux (bottom).

The presence of a time-dependent or external non-conservative force or field drives a system out of equilibrium, thus, detailed balance or time-reversal symmetry is broken. Examples of violations of detailed balance during active processes in biological systems have been reported.^49,85,90–96^ For broken time reversal symmetry, the probability distribution of having a time forward series does not equal the probability distribution of having the time-reversed series. Mathematically, if *P*[{*x*_*τ*_}_0≤*τ*≤*t*_] represents the probability density of having a time-series for a duration of time *t* of a conformational or chemical degrees of freedom (*x*) at time *τ*, then *P̃*[{*θx*_*τ*_}_0≤*τ*≤*t*_] would be the time-reversed probability distribution function, where *θ* = ±1 depending on whether the variable is even or odd under time reversal. For an even variable, like position, *P*[{*θx*_*τ*_}_0≤*τ*≤*t*_] = *P̃*[{*x*_*t*−*τ*_}_0≤*τ*≤*t*_]. Broken time reversal symmetry can also be measured by an asymmetry factor, *A*^*τ*^_*i*,*j*_,^97^ defined by2
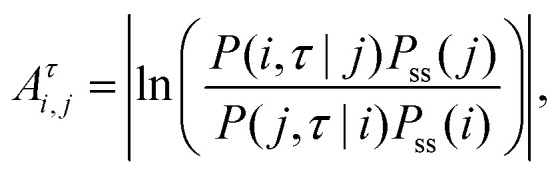
where *P*_ss_(*i*) is the steady state probability density function at state *i* and *P*(*i*,*τ*|*j*) is the conditional probability density that the system is at state *i* at time *τ* given that initially the system was at steady state *j*.

In partially observed systems, some of the net transition fluxes may be obscured, unobserved, or inaccessible. For example, protein copy number inside a living cell as a function of time can be accessed experimentally using live-cell imaging, but whether the corresponding gene is in an active or an inactive state cannot be accessed experimentally.^98^ Although gene activity influences protein copy number, other sources of stochastic noise from transcription and translation also affect copy frequency. As a result, protein copy number alone cannot be used to determine whether a gene is active or inactive. That scenario may lead to an illusion that a nonequilibrium system is at equilibrium, and one needs to rely on the broken time-reversal symmetry in the available, coarse-grained, data to infer irreversibility.

If the coarse-graining commutes with time-reversal symmetry, broken time-reversal symmetry can be quantified *via* the mean-dwell time asymmetry factor (MDAF) for a partially observed system even with a vanishing net current. These systems exhibit second-order semi-Markov statistics.^86^††A stochastic process is Markovian if transitions to future states depend only on the present state. In other words, if the system is currently in state *i*, the probability of transitioning to another state *j* depends solely on being at *i*, and not on previously visited states. In contrast, coarse-graining a Markov process generally induces non-Markovian dynamics, in which the future evolution of the observed system may depend not only on its current state but also on its past trajectory. Such memory-dependent dynamics can often be represented as semi-Markov processes of finite order. In an *n*-th order semi-Markov process, the probability of the next transition may depend on the current state, the time already spent in the current state, and all the previous *n* − 1 visited states. As the order increases, more historical information is required to determine future evolution. For example, in a second-order semi-Markov process, if the system is currently in state *j* and previously visited state *i*, the present state is represented by the ordered pair [*i*,*j*]. The transition from *j* to a subsequent state *k* then depends both on the residence time in state *j* and on the identity of the preceding state *i*. In this framework, the state of the system is represented by an ordered doublet [*i*,*j*], indicating that the system is currently in state *j* and previously visited state *i*. The MDAF is defined as the ratio of the time associated with transitions between doublets of states (*e.g.*, from [*i*,*j*] to [*j*,*k*] for the transition *i* → *j* → *k*) to the time associated with the reverse transition *k* → *j* → *i*.^99–101^Fig. 1(b) illustrates time-reversal symmetry breaking in a system coarse-grained into three discrete states (see inset): 1 (green shaded area), 2 (orange shaded area), and 3 (blue shaded area). The probability distribution of the waiting time at state 2 before transitioning to state 3 in the forward process (1 → 2 → 3) does not coincide with the distribution of the waiting time at state 2 in the reverse process (3 → 2 → 1).

### 2.2 Violation of fluctuation dissipation theorem

Systems at equilibrium follow a fluctuation dissipation relation (FDR) as derived by Kubo,^102^ which links the response of a system to an infinitesimal perturbation to the statistics of its spontaneous equilibrium fluctuations. When a system with an unperturbed Hamiltonian *H*_0_ is perturbed by a force *F*, the perturbed Hamiltonian is given by *H*(**x**,*t*) = *H*_0_(**x**) − *F*(*t*)*A*(**x**), where *A* is the conjugate variable corresponding to the force. The response or the average variation of another generic observable *B* that is coupled to the perturbed observable *A* is given by3
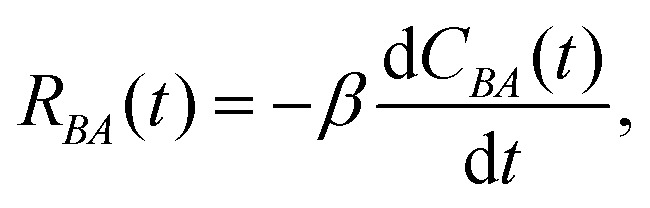
where *R*_*BA*_(*t*) is the response function and *C*_*BA*_ denotes the correlation function between two observables, *A* and *B*, and 
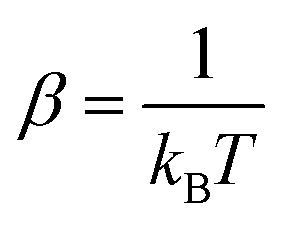
 in the inverse temperature. Eqn (3) is valid for a system at equilibrium. However, a violation of eqn (3) is an indication for an out-of-equilibrium system.

Originally developed within the framework of Hamiltonian statistical mechanics, the FDR has since been generalized to broad conditions, independent of whether the system is Hamiltonian or in equilibrium. There are several other works on the generalization of the fluctuation–dissipation relation for out-of-equilibrium systems.^103–106^ The fluctuation–dissipation theorem (FDT) states that the linear response of a system to an external perturbation can be expressed in terms of the system's equilibrium fluctuations. For systems governed by Langevin dynamics, Kubo showed that the FDT relates the frequency-dependent friction force to the (non-white) random force.^106^ A general linear response formula for a system under time-dependent perturbation was formulated at a NESS, which remains valid far from equilibrium.^105^ A recent review has also discussed FDR in the context of granular systems, nanosystems, and biological systems.^104^

Notably, violation of FDT has been found in a range of systems and processes.^107–112^ Harada and Sasa proposed an equality between the extent of violation of the FDR in the NESS and the rate of energy dissipation into the environment.^113^ Therefore, the violation of FDR had been used to infer the energy dissipation.^113–115^

### 2.3 Non-monotonic time correlation function

Time correlation function of a quantity *Ω* is defined as follows

4 *C*(*τ*) =
〈*Ω*(*t* +
*τ*)*Ω*(*t*)〉

The angular brackets refer to averages with respect to time dependent probability density function, *i.e.*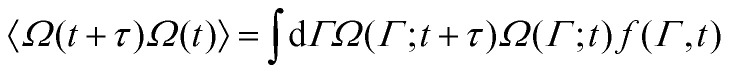, where *f*(*Γ*,*t*) represents the time dependent probability density function of variable *Γ* at time *t*. The decay of a correlation function provides information on whether a system is at equilibrium or far from equilibrium.^87^ The presence of oscillation or non-monotonic decay of correlation function^87^ is an indication of a far-from-equilibrium process, whereas monotonic decay of correlation functions without any oscillations denotes equilibrium. In terms of the eigenvalues of the transition rate matrix,‡‡The off-diagonal elements of the transition rate matrix, *q*_*ij*_, are nonzero and denote the rate of transitioning from state *j* to state *i* per unit time. Each column of the transition rate matrix sums to zero. real eigenvalues signify equilibrium, and complex eigenvalues denote otherwise.^116,117^ Qian *et al.* showed that for a pumped reaction, the eigenvalues are complex.^87^ Similarly, oscillations in the correlation function of the two-state trajectory signify an underlying nonequilibrium system with several microstates lumped into two coarse-grained states.^87^

Let us consider a variable *c* at time *t*. For systems that reach a non-equilibrium steady state (NESS), the time-correlation function 〈*c*(*t*)*c*(*t* + *τ*)〉 exhibits non-monotonic or oscillatory behavior. The angular brackets denote averages over the probability distribution function of the variable at time *t*. A non-monotonic Fig. 1(c) and oscillatory Fig. 1(d) correlation function is an indication for underlying non-equilibrium behaviour. Fig. 1(d) illustrates how concentration fluctuations of two chemical species (namely *x* and *y*) are correlated at time *t*. The normalized correlation function is defined as 
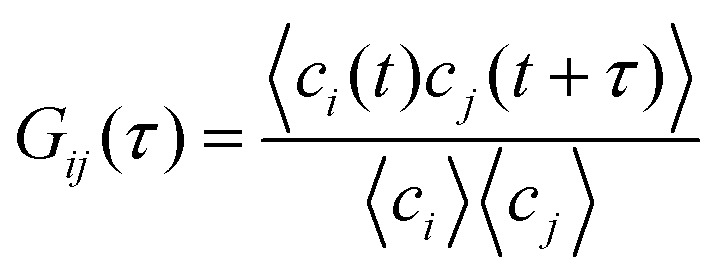
, where *i*,*j* = *x*,*y*,*z*.

### 2.4 Amann–Schmiedl–Seifert criteria and its generalization for NESS

Amann, Schmiedl, and Seifert^118^ identified an alternative criterion for NESS in a three-state system where only one state is observed and the remaining two states are merged into a single mesostate. They proposed a criterion based on observables such as the waiting-time distributions in the observed state and the merged state, as well as the time-dependent probability of being in the observed state. All these quantities can be expressed in terms of the transition rates among the microstates of a given network topology. After rescaling these quantities, conditions leading to negative transition rates are discarded, and violations of the detailed-balance condition are identified as signatures of a NESS. The Amann–Schmiedl–Seifert criterion suggests that if the steady-state probability of occupying state exceeds a certain threshold, the system resides in a NESS. In other words, if the sum of the weights of all spanning trees directed into the ‘on’ state—or equivalently, the time spent in the ‘on’ state—exceeds this bound, then the coarse-grained two-state Markov system will be in a NESS. Therefore, the criterion sets a lower bound for when a three-state system, coarse-grained into two states, can be in a NESS. Notably, the region of parameter space corresponding to a NESS under this criterion is larger than that obtained from the oscillatory correlation function. Subsequently, Jia and Chen^89^ proposed another criterion for NESS based on an observed coarse-grained two-state trajectory arising from an underlying three-state Markov system. They provided a mathematical derivation and a probabilistic interpretation of the Amann–Schmiedl–Seifert criterion, and demonstrated that it also captures non-monotonic or oscillatory correlation functions. Wu *et al.* formulated theoretical framework that detects a nonequilibrium criteria based on coarse-grained observation for a general Markov network with an arbitrary number of microstates and an arbitrary coarse-grained partitioning, given long enough statistics of the trajectory.^119^ The Amann–Schmiedl–Seifert criterion detects a larger NESS region in terms of system parameter values compared to the oscillatory correlation function.^118^

Fig. 1(e), adapted from ref. 89, schematically illustrates the NESS criteria, which shows that the parameter space for a system to be at NESS in terms of the transition rates among the microstates of the full netowrk increases for Amann–Schmiedl–Seifert criterion, and a subset of this parameter space is applicable for the non-monotonic correlation function, and even smaller parameter space is applicable for the oscillatory correlation function. Although, the Amann–Schmiedl–Seifert criterion identifies NESS regimes that cannot be accessed using criteria based on oscillatory or non-monotonic correlation functions, it can also capture the parameter space for NESS which is traced from oscillatory or non-monotonic correlation function.

### 2.5 Other methods

Mori *et al.* showed that if a stochastic process defined by the variable *x* as a function of *τ*, *x*(*τ*), over a time interval [0,*T*] (0 ≤ *t*_m_ ≤ *T*), is at equilibrium, then the probability distribution of *P*(*t*_m_|*T*) would be symmetric with respect to the time *t*_m_ at which *x* reaches its global maximum value.^120^ The area enclosing rate (AER) is another quantity to detect the nonequilibrium nature of a process from two or more particles in a system.^121^ The advantage of this quantity is that it can be computed using only two degrees of freedom.^121^ A three-time-point-position correlation, known as mean back relaxation (MBR), have been recently used to detect broken time reversal symmetry in confinement.^122^ MBR correlates the displacement of a particle between two given time points with its displacement in a prior time period with a finite distance cut-off. For microscopic densities as stochastic observables, the deviation of long time value of MBR from 1/2 is an indication of the nonequilibrium nature of the process.^123^

## 3 Entropy production in fully observed systems

### 3.1 Systems close to equilibrium

Modern thermodynamics provides a unified framework that connects entropy, a central thermodynamic quantity, to irreversible processes occurring in nonequilibrium systems.^12,24,124,125^ In this framework, the total entropy change over an infinitesimal time interval, d*S*, is expressed as the sum of two contributions: one due to exchange with the environment and another due to internal irreversible processes. The exchange term, often written as d_e_*S*, accounts for entropy flow resulting from the transfer of heat or matter, *e.g.*, d_e_*S* = d*Q*/*T* for heat exchange. The second term, d_i_*S*, represents the irreversible entropy production (EP) within the system and is strictly non-negative, in accordance with the second law of thermodynamics.

Irreversible processes such as heat conduction, diffusion, and chemical reactions generate entropy and are characterized as thermodynamic flows driven by corresponding thermodynamic forces. The entropy production rate (EPR), d_i_*S*/d*t*, can thus be formulated in terms of the product of these flows and forces, providing a quantitative description of nonequilibrium dynamics.^81^

### 3.2 EP for systems governed by master equations

In systems described by a continuous-time Markov chain over a discrete set of states, the dynamics are governed by a master equation (ME):^126^5
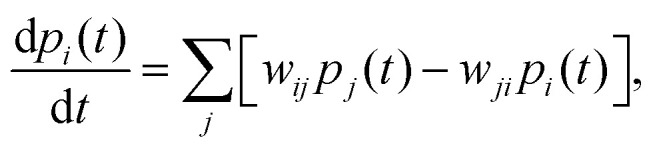
where *p*_*i*_(*t*) is the probability of being in state *i* at time *t*, and *w*_*ij*_ is the transition rate from state *j* to *i*.

For a stochastic trajectory, *γ* = {*i*_0_ → *i*_1_ → ⋯ → *i*_*N*_}, occurring over the time interval [0,*τ*], the total entropy production Δ*S*_tot_ (see Table 1 for a complete list of EP-related symbols) along the trajectory can be written as:6
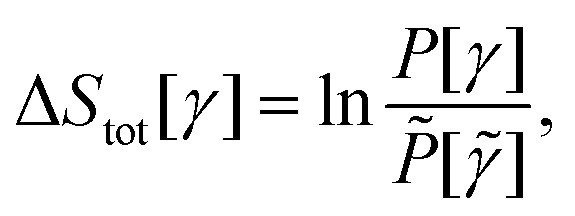
where *P*[*γ*] is the path probability of the trajectory and *P̃*[*γ̃*] is that of the time-reversed trajectory (*γ̃*).

**Table 1:** List of entropy symbols and their definitions

Symbol	Description
Δ*S*_tot_	Total entropy production
Δ*S*_sys_	System entropy production
Δ*S*_m_	Medium entropy production
*σ*	Entropy production rate (EPR)
*σ*_KLD_	EPR estimator based on KLD
*σ*_aff_	EPR estimator based on affinity
*σ*_WTD_	EPR estimator based on waiting times
*σ* ^TM^	EPR estimator for full network
*σ* ^AM^	EPR estimator for approximated master equation
*σ* ^L^	EPR estimator from merging states
*σ* ^SCGF^	EPR estimator using scaled cumulant generating function
*σ*_pp_	Passive partial entropy production estimator
*σ*_ip_ or *σ*^IPEP^	Informed partial entropy production
*σ*_plug_	Plug-in EPR estimator
*σ*_zk_	Zero-knowledge EPR estimator
*σ*_RNEEP_	Recurrent neural network estimator for entropy production
*σ* ^*n*^_tot_	EPR estimator based on an optimization problem considering *n*th moments of waiting times

Expanding the path probabilities yields:7
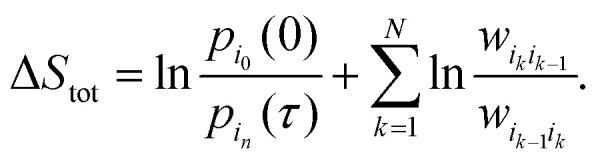


The first term represents the change in system entropy:8
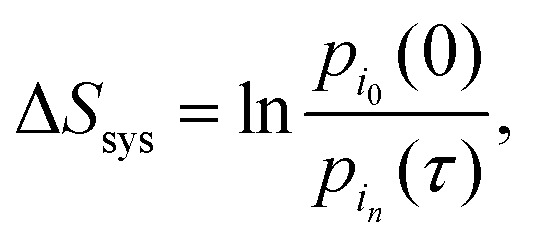
while the second term is interpreted as the entropy flow into the environment:9
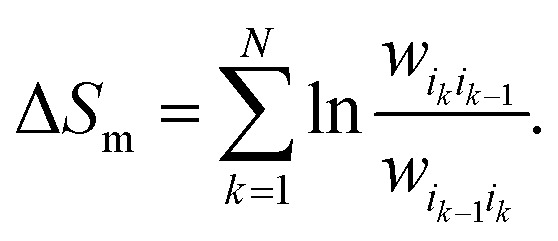


This decomposition implies:

10 Δ*S*_tot_ =
Δ*S*_sys_ +
Δ*S*_m_.

As in the continuous case, this total entropy production obeys the integral fluctuation theorem:^127^

11 〈e^−Δ*S*_tot_^〉 =
1,

which provides a strong statistical constraint on fluctuations in entropy production for finite-time, nonequilibrium trajectories. It also ensures consistency with the second law: 〈Δ*S*_tot_〉 ≥ 0.

Below, we derive the calculation of the EPR rate for a system following Markovian statistics.^128^ We consider a trajectory *γ* with a sequence of *N* states {*i*_0_,…,*i*_*N*_} and the corresponding waiting times {*t*_0_,…,*t*_*N*_} for a total observation time *T*, 
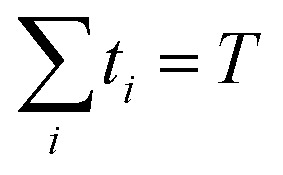
, given by *γ* = {(*i*_0_,*t*_0_),…,(*i*_*N*_,*t*_*N*_)}. The probability of observing such a trajectory with initial probability distribution π_*i*_0__ is given by12
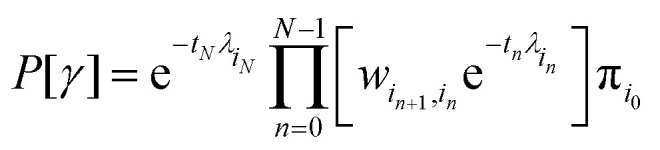
where *λ*_*i*_ is the escape rate from state *i*, 
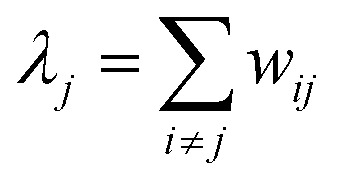
.

Similarly, one can define the probability *P̃*[*γ̃*] for the time-reversed trajectory *γ̃* = {(*i*_*N*_,*t*_*N*_),…,(*i*_0_,*t*_0_)}, and express the total EP along the trajectory, Δ, using their ratio:^129^13
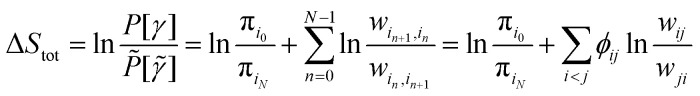
where *ϕ*_*ij*_ is the net number of transitions from state *j* to state *i*.

The EPR can be calculated from the evolution of the EP governed by the Master equation. Following Teza and Stella,^78^ we denote the probability *P*_*i*_(*S*,*t*) that the system is in state *i* at time *t*, having produced entropy *S* from all possible trajectories up to the time *t*. Since each transition between states *i* and *j* adds ln(*w*_*ji*_/*w*_*ij*_) to the total EP, the Master equation for *P*_*i*_(*S*,*t*) reads:14
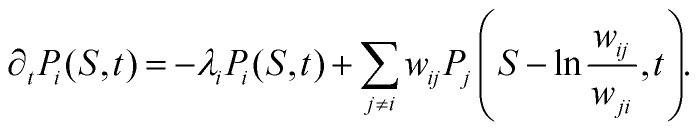


Let us denote15
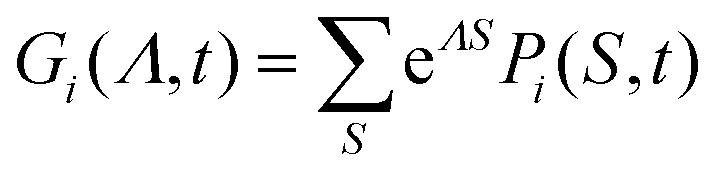
as the discrete Laplace transform of *P*_*i*_(*S*,*t*) with respect to *S*. Eqn (14) then becomes:16
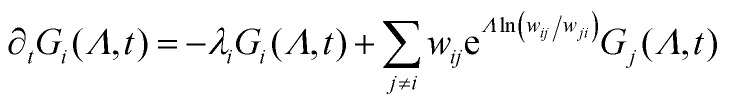


In matrix form,

17 ∂_*t*_*G*(*Λ*,*t*)
= *W̃G*(*Λ*,*t*)

where *G*(*Λ*,*t*) is a column vector with *G*_*i*_(*Λ*,*t*) as its *i*-th entry, and *W̃* is the tilted transition matrix:^78,130^18
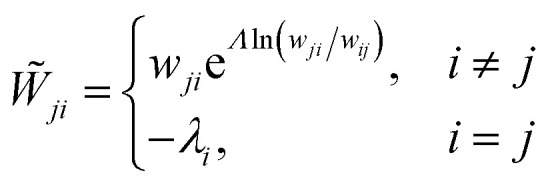


The dominant eigenvalue *Ω*^TM^ of *W̃* is the scaled cumulant generating function (SCGF) of the entropy production. The mean EPR is given by:^78,130,131^19
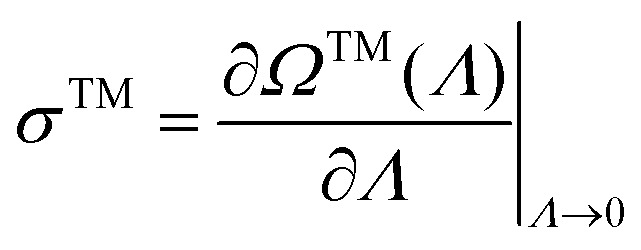


Here, TM refers to “Total Mean”, indicating that all state dynamics are considered.

### 3.3 EP along a stochastic trajectory

For systems described by overdamped Langevin dynamics, the evolution of the probability density (*p*(*x*,*t*)) is governed by a Fokker–Planck equation (FPE). Within the framework of stochastic thermodynamics, the entropy production^136^ associated with a single trajectory can be decomposed into two components: the change in system entropy and the entropy flow into the surrounding medium.^81,124^ The system entropy at time *t* is defined as^23,124^

20 *S*_sys_(*t*) =
−ln *p*(*x*(*t*),*t*),

where *x*(*t*) is the system's state along a stochastic trajectory, and *p*(*x*(*t*),*t*) is the instantaneous probability density at time *t*.

The entropy change of the medium, often associated with the heat dissipation into the environment (assumed to be at constant temperature), is given by21
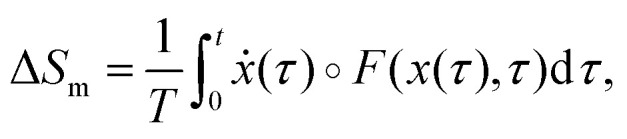
where *F*(*x*,*t*) is the total time-dependent force, which can result from a combination of a conservative potential and a non-conservative force, *ẋ* is the velocity along the trajectory, and ∘ is the Stratonovich product.^124^ The total EP along a single trajectory as defined by eqn (10), satisfies the integral fluctuation theorem, eqn (11),^127^ which holds for arbitrary initial conditions and time-dependent driving. This result generalizes the second law of thermodynamics to the level of individual stochastic trajectories and highlights the inherent irreversibility of nonequilibrium processes, even in the presence of thermal fluctuations.

### 3.4 Fully observed systems

**Fig. 2:**
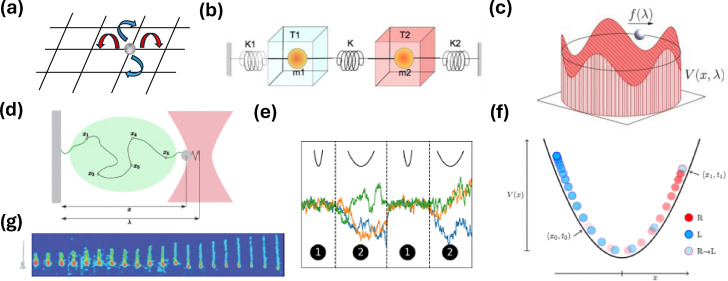
Schematic of systems with full accessibility of the dissipative degrees of freedom. (a) Random walk on a two-dimensional lattice. (b) Two Brownian oscillators of masses *m*_1_ and *m*_2_ are bound by harmonic potentials of strengths *k*_1_ and *k*_2_, respectively. The oscillators are coupled by a harmonic potential of strength *k*, and each is in contact with a heat bath, one at temperature *T*_1_ and the other at temperature *T*_2_.^132^ This figure has been reproduced from ref. 132 with permission from the IOP Publishing, copyright 2026. (c) The state of a colloidal particle is defined by its position *x*; it is driven by a periodic potential *V*(*x*,*λ*) and a non-conservative force *f*(*λ*), where *λ* is a control parameter. This figure has been reproduced from ref. 23 with permission from the IOP Publishing, copyright 2026. (d) Schematic of polymer pulling using an optical trap: one end of the macromolecule is fixed to a surface, while the other end is attached to a colloidal particle pulled by an optical trap. The distance between the bead and the fixed surface (*λ*) is varied. This figure has been reproduced from ref. 23 with permission from the IOP Publishing, copyright 2026. (e) Trajectories of three diffusing particles driven by harmonic potentials of varying stiffness, shown in different colors. This figure has been reproduced from ref. 133 with permission from the American Physical Society, copyright 2026. (f) Run-and-tumble particle. This figure has been reproduced from ref. 134 with permission from the IOP Publishing, copyright 2026. (g) Experimental images of polymer extension in a hydrodynamic flow field. This figure has been reproduced from ref. 135 with permission from the Royal Society of Chemistry, copyright 2026.

The methods described in Sections 3.2 and 3.3 can provide the total EP if all dissipative degrees of freedom are known. Full EP has been calculated in various systems, including particles exhibiting overdamped Brownian dynamics,^137^ active Brownian particles (ABPs) under velocity-dependent active forces,^138^ coupled harmonic oscillators connected to heat baths at two different temperatures,^132,139^ Brownian duets,^140^ Brownian particles in moving traps,^141^ particles undergoing underdamped Langevin dynamics with active forces, both with and without confinement,^142^ harmonically dragged Brownian particles with time-varying stiffness,^143,144^ harmonically bound particles under time-dependent forces,^145^ polymers in hydrodynamic flow fields,^146^ run-and-tumble particles diffusing in harmonic potentials,^134^ and electric circuits.^147–149^Fig. 2 shows examples of systems with various driven degrees of freedom.

## 4 EP from partial information

### 4.1 Partially observed system

As discussed in Sections 3.2 and 3.3, inferring dissipation in terms of EP requires all relevant mesostates to be experimentally accessible with thermally equilibrated fast hidden dynamics on the microstates. However, due to finite spatiotemporal resolution, all relevant mesostates might not be accessible, and therefore, we obtain a coarser level description of the system. In practice, finite spatiotemporal resolution yields a coarse-grained description in the absence of timescale separation between the microstates. The unresolved microscopic/mesoscopic degrees of freedom render the observed dynamics non-Markovian. Therefore, residence times in the observable states for partially observed systems are no longer exponentially distributed.

**Fig. 3:**
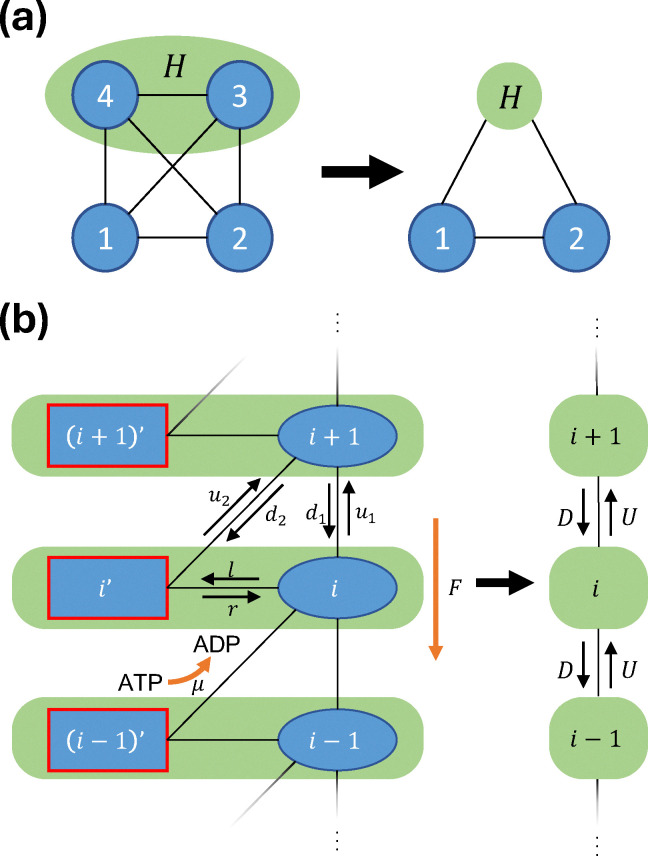
Fully resolved (left) and coarse-grained (right) representations of two model systems: (a) schematic shows a 4-state fully connected network where the discrete states are denoted by circles with numbers 1, 2, 3, and 4, and transitions between them are represented by solid lines. States 1 and 2 are observed and states 3 and 4 are merged together into a lumped state *H*; (b) schematic of a fully resolved (left) and a coarse-grained (right) model of a molecular motor. The discrete states representing the position of the molecular motor are represented by the numbers *i* − 1, *i*, and *i* + 1, where at each position, the motor can be active (red rectangles) or passive (ellipses). Only the position of the motor is accessible to an observer, while whether it is active or passive cannot be resolved. As such, it is a partially observed system. This figure has been reproduced from ref. 150 with permission from the American Physical Society, copyright 2026.

A model example of a partially observed system is a fully-connected four-state system, in which two of the states cannot be resolved and are therefore coarse-grained into a single hidden state (Fig. 3(a)).^129,151^ Specifically, two states remain as observable (Markovian) states, while the other two are combined into one macrostate. An external control parameter is introduced to tune the transition rates across the observed link, thereby influencing the observed dynamics. The transition rates between state 1 and 2: *w*_12_(*F*) = *w*_12_e^−*βFL*^ and *w*_21_(*F*) = *w*_21_e^*βFL*^, where *β* = *T*^−1^ is the inverse temperature (with *k*_B_ = 1), *F* is the applied force and *L* is a characteristic length scale.

Fig. 3(b) schematically shows another model system that serves as an example of a partially observed process. It depicts a molecular motor that moves along a one-dimensional track in discrete steps, either “up” or “down.” At each spatial position, the motor can exist in either an active or a passive internal state. As illustrated in the figure, the system can undergo spatial transitions between neighboring positions (*i* ↔ *i* + 1) or switch between internal passive and active states (*i* ↔ *i*′). In the active state, upward transitions are favored by a chemical potential difference Δ*μ*, while an external force (*F*) acts downward, opposing this preferred direction. We assume that an external observer cannot distinguish between the active and passive internal states and can only record the motor's position. As a result, the observed dynamics reduce to a second-order semi-Markov process, which can be represented as a three-state cyclic network, where each state corresponds to a physical location that combines the active and passive substates. The transition rates satisfy local detailed balance, with (Δ*μ*) affecting only the active-state transitions and (*F*) influencing all spatial transitions, such that 
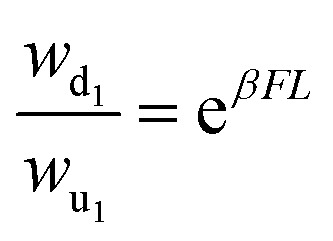
 and 
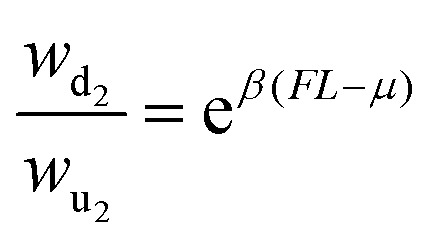
.

**Fig. 4:**
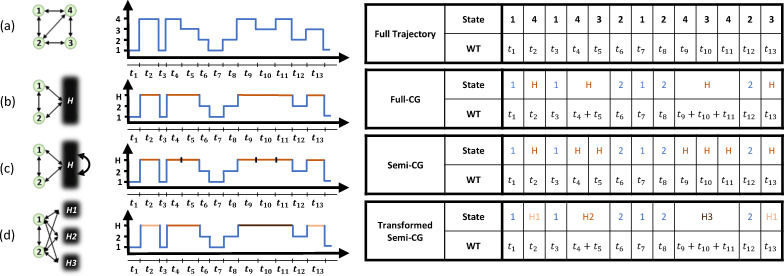
Illustration of partial-information frameworks for a four-state system. (a) Fully observed system, with trajectories specified by microstates labeled 1, 2, 3, and 4, and the waiting times (WTs) spent in each state before transitioning to the next state. (b) Full coarse-graining (full-CG): states 3 and 4 are merged into a single mesostate labeled *H*. (c) Semi-coarse-graining (semi-CG): states 3 and 4 remain spatially indistinguishable, but transitions between them can be recorded, with each WT measured for the hidden microstates. (d) Transformed semi-CG: every *n* consecutive visits to the hidden states are grouped into *H*_*n*_, with the WT given by the sum of the WTs over the *n* visits. This figure has been reproduced from ref. 152 with permission from the American Physical Society, copyright 2026.

Theoretical developments in stochastic thermodynamics employ different coarse-graining methods to consider partial information that mimics experimental spatiotemporal limitations. We now introduce two types of coarse-graining (CG) approaches in ref. 152, as shown in Fig. 4. In the full-CG approach, a subset of microstates is lumped into a single observed state, giving rise to a second-order semi-Markov process, since the waiting time in the hidden state depends on the previously visited state. In this example, states 1 and 2 are observed, whereas states 3 and 4 cannot be distinguished and are recorded as a single state *H*. The waiting time in *H* is then the sum of the corresponding waiting times in microstates 3 and 4 before transitioning to one of the observed states. In the semi-CG scheme, we assume that an observer can record intra-transitions within the hidden states (Fig. 4(c)). For example, a sequence 1 → 4 → 3 → 2 is recorded as 1 → *H* → *H* → 2, with the corresponding waiting times (*i.e.*, the time spent during the first and second visits to *H*) recorded separately. In this case, although the initial and final microstates are not distinguished because they are both lumped into same macrostate, the additional information can still be exploited to improve the lower bound on the total EPR.

Van der Meer *et al.* developed a method to infer entropy production in partially observable Markov systems by analyzing observable transitions and the waiting times between them.^153^ Using ratios of waiting time distributions, they built an estimator that either recovers the full entropy production (when no hidden cycles exist) or provides an improved lower bound when hidden cycles are present. They also showed that these waiting-time statistics reveal hidden network features such as cycle presence, length, and affinity. By formulating the problem in an equivalent semi-Markov framework, they unified earlier entropy estimators under a fluctuation-theorem perspective and clarified the role of the correct time-reversal operation.^153^

Around the same time, Harunari *et al.* developed a framework for inferring dynamical and thermodynamic properties from systems where only a subset of transitions is observable.^154^ They derived analytic expressions for the probabilities and timing of successive visible transitions and used these to build a lower bound on entropy production that remains informative even when no net currents are observed. They showed that repeated and alternated transition statistics carry distinct physical information, allowing one to detect irreversibility or hidden disorder in the underlying network. Their approach was validated with numerical simulations and demonstrated its applicability to experimentally motivated models such as molecular motors, highlighting the broader power of transition statistics for probing partially accessible Markov processes.^154^

### 4.2 EPR from partial information

Evaluating the total EP requires full knowledge of all the underlying system dynamics, which is not always accessible. However, partial EPRs can be obtained from the observed information. For these trajectory observables, the auxiliary generator is obtained by modifying the hidden transitions.

The passive partial EP is defined by modified transition rates as follows:^129,155^22
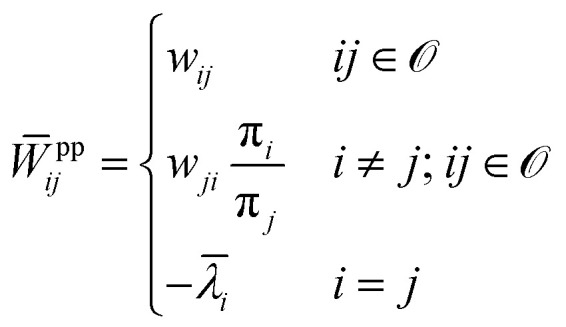
where pp in *W̄*^pp^_*ij*_ stands for “passive partial”. The observed substates form a set denoted by 𝒪. *w*_*ji*_ and π_*j*_ denote the transition rate from *i* to *j* and the steady state probability for the state *j*, respectively. The steady state probabilities are obtained from **W**π = 0, and also 
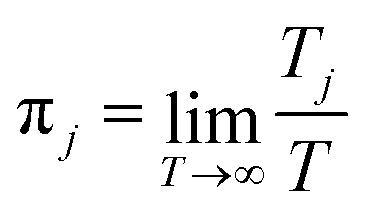
 (the fraction of time spent at each state at long time limit). *λ̄*_*i*_ denotes exit rate from state *i* and ensures probability conservation (see ref. 129 for the analytical expression of *λ̄*_*i*_).

The other partial EP is known as the informed partial entropy production, and it is defined by the following:23
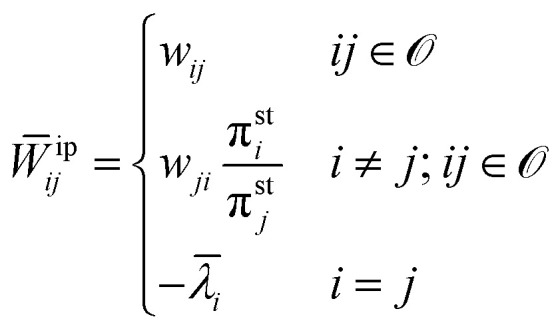
where ip in *W̄*^ip^_*ij*_ stands for “informed partial”. π^st^_*j*_ refers to the stall probability distribution for which the flux between the observed substates vanishes. The stall probability distribution satisfies *w*_12_(*x*^st^)π_2_(*x*^st^) = *w*_21_(*x*^st^)π_1_(*x*^st^), where *x*^st^ is the value of the control parameter that stalls the observed current. Note that in both cases of the passive and informed partial entropy production, the entries of the auxiliary rate matrices, *W̄*^pp^ and *W̄*^ip^, are identical to the ones of original rate matrix *W* for the observed transitions *ij* ∈ 𝒪, where the hidden transitions are multiplied by ratios of either the state-state probabilities or the stalling probabilities, respectively. This formulation gives rise to the development of fluctuation theorems for both partial entropy productions from a unifying perspective.^129^

The passive partial entropy production rate (PPEP), *σ*_pp_, is an estimator of the total EPR calculated from the transitions between two observed states, which provides a lower bound on the full entropy production.^129^ Suppose we observe only two states *i* and *j*, that form Markovian subsystems and record the transitions between them. In this case, we can compute the transition fluxes or transition rate times the steady state probability density *n*_*ij*_ = *w*_*ij*_π_*j*_ and *n*_*ji*_ = *w*_*ji*_π_*i*_, and use them to evaluate the EP inferred from these transitions:24
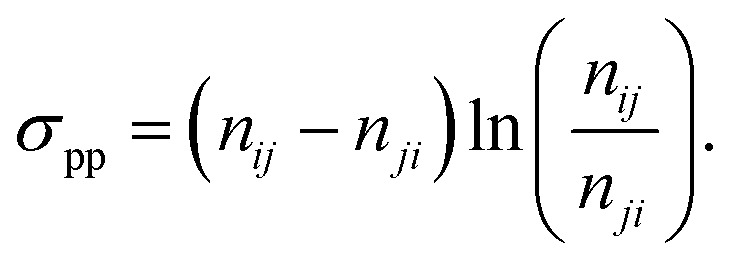


The average informed partial entropy production rate (IPEP), *σ*_ip_, is given by25
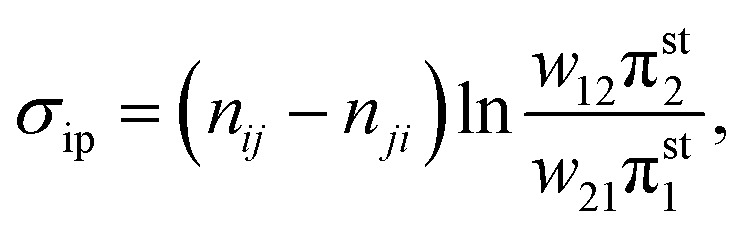
where only states 1 and 2 are observed. Like passive partial EP, informed partial entropy production provides alower bound on the total entropy production. PPEP or IPEP and their corresponding complementary parts sum up to the total entropy production rate. Contribution to the complementary parts of the EP arises from the hidden part of the network. Both PPEP and IPEP satisfies the detailed fluctuation theorem.

Ehrich obtained a tight lower bound on the entropy production from dynamics of a hidden Markov model that is compatible with the observed data.^156^ Shiraishi *et al.* showed that the partial entropy production satisfies the integral fluctuation theorem for both autonomous and non-autonomous nanomachines.^155^

### 4.3 Coarse-grained (space) EPR

The amount of information lost describing a partially observed physical system would depend on the coarse-graining procedure. Therefore, recovering the total dissipation from imperfection of partial information and role of finite spatial resolution and finite statistics on the dissipation estimation has attracted a lot of attention in recent times.

Busiello *et al.*^157,158^ investigated EP in nonequilibrium systems by comparing stochastic dynamics described by a Master equation (ME) with their coarse-grained representation *via* FPE. It showed that coarse-grained EP contains contributions from microscopic probability currents that are absent in the FPE description and therefore cannot be recovered from it. By comparing ME and Fokker–Planck representations for discrete- and continuous-state systems, the authors derived analytical corrections to the entropy production and identified conditions under which no information is lost in the coarse-graining when moving from an ME to an FPE. The findings, which were experimentally testable, offer a method to infer hidden microscopic processes from coarse-grained measurements.^157,158^

Generally, coarse-graining reduces the complexity of a system either by merging states or by eliminating them. State-merging approaches, including lumping and milestoning, combine multiple microstates into a single mesostate. In contrast, state-elimination methods, such as trimming and decimation, remove selected states from the original network. State-merging methods may reflect experimental systems observed with finite spatiotemporal resolution or systems exhibiting timescale separation, where states connected by fast transitions are grouped into an effective state. State-elimination methods, on the other hand, may correspond to experimental situations in which certain states are unobserved. Whether trajectory functionals of the coarse-grained system remain identical to those of the full system depends on the specific coarse-graining procedure employed.^153^

**Fig. 5:**
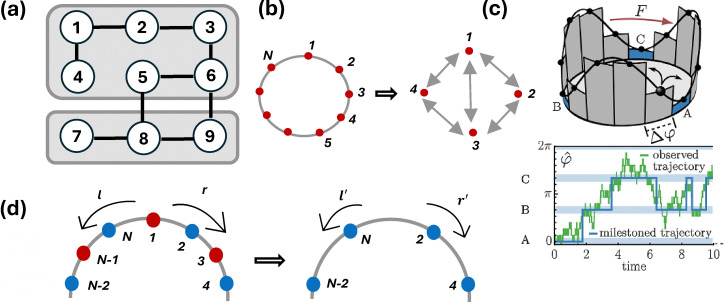
Schematic of different coarse-graining methods. Lumping and milestoning are state-merging coarse-graining methods, whereas decimation and trimming are state-eliminating coarse-graining methods. (a) Lumping of a discrete Markov chain, in which multiple substates are merged into a single mesostate. (b) Trimming, where states are progressively eliminated from the network while keeping the transition flux among the remaining states unchanged. This figure has been adapted from ref. 79 with permission from the American Chemical Society, copyright 2026. (c) Milestoning – another state-merging coarse-graining method – in which the system remains in a coarse-grained (“milestoned”) state until its system trajectory reaches another milestoned state. This figure has been reproduced from ref. 159 with permission from the National Academy of Sciences, copyright 2026. (d) Decimation, a state-eliminating coarse-graining approach in which selected states are removed from the network. In this schematic, odd-numbered states are eliminated. This figure has been adapted from ref. 78 with permission from the American Physical Society, copyright 2026.

State lumping is a widely used approach for coarse-graining, in which several microstates are coarse-grained into mesoscopic states as shown in Fig. 5(a).^160^ Recently, Igoshin, Kolomeisky, and Makarov have proposed a state eliminating coarse-graining method called trimming^79^ (Fig. 5(b)) that preserves the mean EPR in the absence of a dissipative cycle. This coarse-graining method eliminates states and reassigns the transition rates determined by splitting probabilities and mean first passage times. The observed dynamics of the remaining microstates that do not undergo decimation remain unchanged after the CG method. However, this method might not preserve the network topology for eliminating any state with more than two connections. In this method, the dynamics of the coarse-grained system follow a semi-Markov process, with nonexponential distributions of waiting times between jumps, and it does not require any knowledge about the true microscopic dynamics.

Milestoning^159,161^ is another coarse-graining framework (Fig. 5(c)) in which the continuous state space of a stochastic dynamical system is partitioned into a set of milestones, *i.e.*, carefully placed, low-dimensional hypersurfaces that delineate metastable regions or “cores”. Instead of tracking the full microscopic trajectory, milestoning records only the sequence of milestone crossings and the statistics of transition times between them.

By stitching together these short trajectory segments, one can compute rate constants and estimate thermodynamic quantities. If the equilibration time in each milestoned state is short compared to the excursion times (time spent in between visiting the milestoned states), the system may be approximated as Markovian.^161^

Blom *et al.*^159^ clarify the fundamental difference between coarse-graining by lumping and by milestoning. Lumping maps many microscopic configurations onto broad observable states, which typically introduces memory and results in non-Markovian dynamics even when the underlying system is Markov. Milestoning applies an additional postprocessing step. Instead of tracking the system whenever it occupies a lumped state, it records only first passages between selected milestones. This filtering eliminates repeated visits within the same region and often yields a low-order semi-Markov process that, under some conditions, may bring the observable dynamics closer to the underlying irreversibility.

Decimating states (for example, odd numbered states are decimated in Fig. 5(d)), as mentioned by Pigolotti and Vulpiani^162,163^ approximate the dynamics of the coarse-grained system by a Markovian master equation. This method eliminates short-lived states by assuming that these are in a quasi-steady state. The probabilities of being in short-lived states can then be expressed as those for the remaining states, resulting in an effective approximate dynamic description that does not explicitly consider the short-lived states. Subsequently, it was demonstrated that in the presence of time-scale separation, the decimation of fast states does not affect entropy production estimates. In trimming, jumps between observed states are counted, whereas jumps between an unobserved state and an observed state are not counted. This distinguishes trimming from the decimation method.

Teza *et al.*^78^ showed that memoryless jump processes can be coarse-grained (Fig. 5(d)) while exactly preserving the stationary average and fluctuations of EP. This method assumes complete knowledge of the underlying microscopic dynamics. It eliminates a subset of states from the ME for the probability of the system being in state *i* at time *t*, and expresses the entropy *S* in terms of non-normalized probabilities of the remaining states, which are sufficient to exactly recover the long-time average EPR. This CG method assumes the knowledge of microscopic dynamics before coarse-graining for feasibility of ME formulation.

Bilotto *et al.* studied how coarse-graining affects on entropy production by considering a model system of a one-dimensional colloidal particle in contact with a thermal bath under a sinusoidal potential and driven out of equilibrium by a small constant force.^164^ The authors found that at large friction coefficients, both underdamped and overdamped dynamics yield identical entropy production. In contrast to that, at smaller friction, the overdamped approximation overestimates entropy production compared to the underdamped case. By approximating the continuous dynamics of a colloidal particle under periodic potential as a Markov chain model *via* spatio-temporal discretization, the entropy production at underdamped and overdamped limits for varying friction coefficients was also studied. The authors found that at a small friction limit, inertia plays a significant role in the loss of entropy production in small friction coefficient regime and gain of entropy production in a regime of intermediate frictions.^164^ Another instance where the actual EP turns out to be lower than the CG entropy production estimated in ref. 165, where the authors studied a kinetic network with fast-pumping dynamics and slow-network dynamics.

In a coarse-grained description, we often integrate out bath degrees of freedom. However, whether this CG would affect the entropy production rate of the probe particle depends on whether the integrated-out degrees of freedom are dissipative or non-dissipative. Busiello *et al.* employed a generalized Langevin equation (GLE) to model the dynamics of a probe particle immersed in an active bath.^166^ The authors found that if the active bath degrees of freedom interact with the probe particle non-reciprocally, then eliminating those entropic degrees of freedom would result in a change in the entropy production. However, for elimination of non-entropic degrees of freedom, there would by no effect on the EPR estimates.^166^ Yu *et al.*^167^ showed that CG EPR estimates followed a power law relation with the spatial resolution and highlighted the importance of accounting for the correlation between flux at lower resolutions.

### 4.4 Coarse-grained (time) EPR

Yu *et al.*^167^ found that CG EPR is non-monotonic in temporal resolution, with its peak position revealing characteristic timescale corresponding to the underlying dissipative process. This observation is also found in the actomyosin cortex of starfish oocytes.^168^ This method provides characteristic dissipative scales from dissipation measurements.^169^ In a recent study, Fritz *et al.* investigated the effect of EPR estimators based on waiting times and TUR on finite temporal resolution, assuming that all observed transitions are registered.^170^ They found that the waiting-time estimator based on resolved transitions performs best at finite temporal resolution, given perfect measurement statistics.

## 5 Quantifying degree of irreversibility

In this section, we discuss theoretical tools for estimating dissipation in non-invasive ways that can be directly applied to experimental data. Quantifying dissipation is crucial because a non-zero steady entropy production rate not only characterizes a nonequilibrium process in biological and artificial systems,^12,23,124,171^ but also determines the “biological quality”^172^ or efficiency of energy transduction in microscopic machines.^173–177^ Moreover, dissipation constrains precision in sensory adaptation,^178,179^ the regularity of biological clocks,^172^ and several other fundamental processes.

As we will see in this section, estimating the probability distribution or the probability current is essential for quantifying entropy production. However, obtaining the probability distribution depends on the underlying system dynamics, and the probability current is not always experimentally accessible or theoretically known without the dynamical equations. In such cases, it is necessary to estimate entropy production using model-free approaches. These approaches will also be discussed in the following subsections.

### 5.1 EPR calculation from KLD

Kullback–Leibler divergence (KLD) quantifies the distinguishability between two probability distributions,26
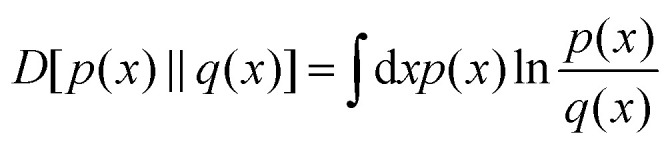


KLD is a positive quantity that vanishes only when the probability densities are equal, *p*(*x*) = *q*(*x*) for all *x*. If an experiment measures a single random variable at a finite frequency, one obtains a discrete trajectory (say length *n*) as an output which can be written as *x* = {*x*_1_,*x*_2_,*x*_3_,*x*_4_,…,*x*_*n*_}. However, if we do not know the true distribution (say *Q*) from which *x* is sampled, and incorrectly guess it as *P*, then the KLD quantifies the amount of information lost when the true probability distribution *Q* is approximated by another distribution *P*.^180^

If *P*(*x*) and *Q*(*x*) represent probability distributions for time-forward and time-backward series or phase space densities, then KLD measures the irreversibility or relative entropy.^181^ In that scenario, the expression for average entropy production rate, 〈*Ṡ*〉, at NESS (*τ* → ∞) becomes^182^27
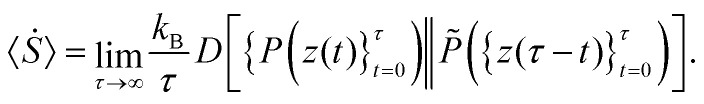


Eqn (27) applies for variables with even parity. KLD between phase space densities of the forward (*ρ*) and backward (*ρ̃*) transitions for systems transitioning between two equilibrium states is related to the average dissipated work,^183^

28 〈*W*_diss_〉 =
*k*_B_*TD*(*ρ*‖*ρ̃*).

*k*_B_ is the Boltzmann constant and *T* is the temperature of the heat bath where the system relaxes to a steady state. The average dissipated work is the difference between the average work, 〈*W*〉, and the equilibrium free energy change, Δ*F*, *i.e.*, 〈*W*_diss_〉 = 〈*W*〉 − Δ*F*. *D*(*ρ*‖*ρ̃*) in eqn (28) is called the relative entropy.^184^ Total entropy production was found to be related to the distinguishability of a time forward to its time-reversed process, quantified by the relative entropy between forward and backward states.^181^ In other words, the total entropy production is the dissipated work divided by temperature,^181^

29 *S* =
〈*W*_Diss_〉/*T* =
*k*_B_*D*(*ρ*‖*ρ̃*)

Eqn (29) applies to different initial states and both classical and quantum systems. The dissipated work can also be calculated from the forward and reverse work distribution (*P*_F_ and *P*_B_, respectively) where the protocol is reversed for obtaining the reverse work distribution, 〈*W*_Diss_〉 = *k*_B_*D*(*P*_F_(*W*)‖*P*_B_(*W*)).^182^

The zero-knowledge estimator, *σ*_zk_, does not assume any dynamical model, and calculates the EPR from the observed dynamical activity and observed probability distribution of the observed transitions using the formula:^185^30
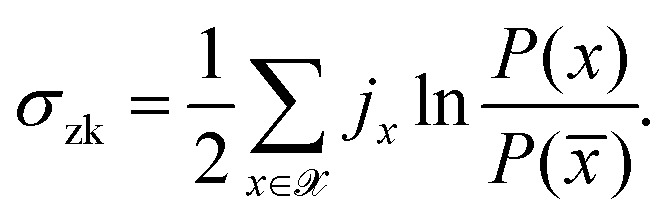


The zero-knowledge EPR estimator *σ*_zk_ requires the observed transition flux, *j*_*x*_ = 𝒦_obs_(*P*(*x*) − *P*(*x̄*)) (𝒦_obs_ being the dynamical activity or total number of events per unit time), the unconditional probability distribution of the observed event *P*(*x*) and its time-reversed counterpart, *P*(*x̄*). Ref. 185 studies multifilar events and showed *σ*_zk_ provides a lower bound on the total dissipation.

KLD is a versatile tool for calculating several thermodynamic quantities,^182,186^ and several KLD estimators exist, such as the plug-in method^187^ and estimators based on compression algorithms.^188^ The plug-in estimator, *σ*_plug_, was introduced to approximate the Kullback–Leibler divergence (KLD) between forward and reverse sequences of discrete stationary time series by counting data sequences and estimating their probabilities.^182,189^ The *m*th-order approximation of the KLD between sequences of length *m* is:31
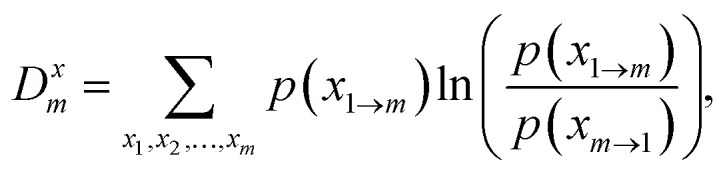
where *p*(*x*_1→*m*_) and *p*(*x*_*m*→1_) denote the probabilities of observing the forward sequence *x*_1→*m*_ = (*x*_1_,…,*x*_*m*_) and the corresponding backward sequence *x*_*m*→1_ = (*x*_*m*_,…,*x*_1_), respectively. These probabilities can be estimated from the frequency of each sequence in a sufficiently long trajectory. The incremental slope of *D*^*x*^_*m*_ as a function of *m*,^182^

32 *d̂*^*x*^_*m*_
= *D*^*x*^_*m*_ − *D*^*x*^_*m*−1_,

converges to the entropy production per step in the limit of large *m*. Non-Markov processes can be represented as a semi-Markov process of any order, however, calculating *d̂*^*x*^_*m*_ becomes difficult for large *m*. To address this, the following ansatz^182^ has been proposed:33
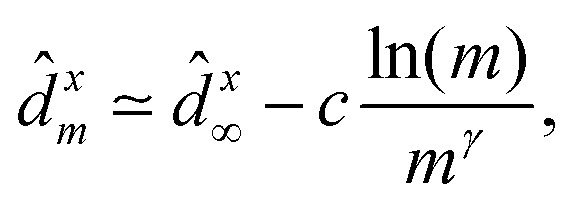
where *d̂*^*x*^_∞_, *c*, and *γ* are fit parameters for *d̂*^*x*^_*m*_ as a function of *m*.

The plug-in estimator for the entropy production rate per unit time is then given by34
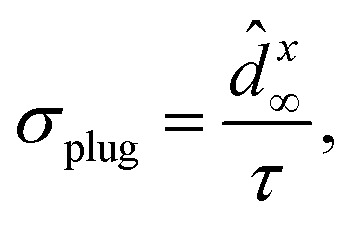
with *τ* denoting the mean waiting time per step. We applied this estimator to a partially observed four-state system, as well as to molecular motor and flashing ratchet models; the results are discussed in Section 5.7.^152^

Eqn (28) and (29) hold only when the full phase-space density or all driven degrees of freedom are accessible. For coarse-grained descriptions, eqn (29) reduces to^190^

35 〈*W*_diss_〉 ≥
*k*_B_*TD*(*ρ*‖*ρ̃*),

With partial information or finite-length trajectories, KLD provides only a lower bound on entropy production. In this case, eqn (29) for a trajectory of length *t* becomes^191^36
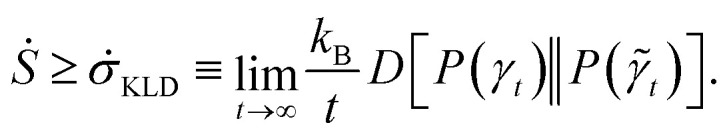


A hierarchy of inequalities can then be established for *k*-variable irreversibility:^192^

37 0 ≤ *S*_1_ ≤
*S*_2_ ≤ ⋯ ≤ *S*_*k*_ ≤
⋯ ≤ *S*_tot_.

where the subscript *k* of *S*_*k*_ denotes the number of driven variables considered when evaluating the KLD.

Partially observed systems generally lose their Markovian character but can be modeled as semi-Markov processes. In a second-order semi-Markov process, the observed states are reformulated as doublets, [*ij*], where the first index denotes the previous state and the second the current state. For such processes, the KLD-based estimator of entropy production, *σ*_KLD_, can be decomposed into two contributions,^99^

38 *σ*_KLD_ =
*σ*_aff_ +
*σ*_WTD_,

where *σ*_aff_ corresponds to entropy production from state affinities, and *σ*_WTD_ arises from asymmetries in the waiting-time distributions (WTDs). The affinity contribution is given by^99^39
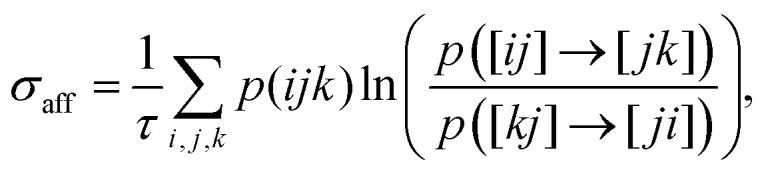
where *p*(*ijk*) is the probability of observing the sequence *i* → *j* → *k*, expressed as^99^

40 *p*(*ijk*) =
*p*([*ij*] →
[*jk*])*R*[*ij*],

with *p*([*ij*] → [*jk*]) the probability of a transition *j* → *k* given *i* → *j*, and *R*[*ij*] the fraction of visits to the state pair [*i*,*j*]. Thus, *σ*_aff_ is determined by the ratio of forward to backward transition probabilities.

The WTD contribution stems from the KLD between forward and backward waiting-time distributions:^99^41
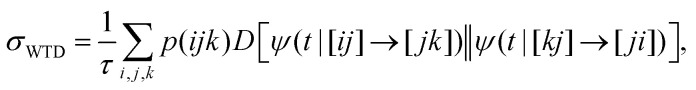
where *ψ*(*t*|[*ij*] → [*jk*]) is the WTD in state *j*, conditioned on the previous state *i* and subsequent state *k*, and *τ* is the mean waiting time per state.

Eqn (41) is valid for second-order semi-Markov processes when coarse-graining is local in time and commutes with time reversal.^100,101^ One such example was demonstrated in ref. 86, which we discuss below. The entropy production was inferred for an experimental partially observed system of hair-cell bundle oscillations, driven by molecular motors.^86,193,194^ For this system, the tip position of the hair bundle is accessible, whereas the positions of the molecular motors remain hidden. We estimated the irreversibility of such partially observed oscillatory dynamics, governed by coupled overdamped Langevin equations. However, estimating entropy production rates in continuous-variable systems was challenging due to finite spatiotemporal resolution and the limited accessibility of all driven variables. To address this, we coarse-grained the observed variable of the nonequilibrium system into a few discrete states and estimated a lower bound on the total entropy production rate (EPR) from the Kullback–Leibler divergence (KLD) between waiting-time distributions (WTDs), exploiting the underlying time irreversibility.^195^ We further proposed the mean dwell-time asymmetry factor, defined as the ratio between the mean dwell times along the forward and backward directions, as an estimator of EPR. This quantity provided a qualitative measure of broken time-reversal symmetry and increased with finer spatial resolution. We applied this estimator to the above-mentioned example of a second-order semi-Markov process.

EPR inference for partially observed systems was done using the coarse-graining approach as discussed in Section 4. Additional approaches rely on observed transitions and waiting time distributions.^153,154,196,197^ Dominic *et al.* introduced a bound on the EPR from the waiting time statistics of hidden Markov processes. They further used their estimator to quantify the irreversibility in various biological processes like in gene regulatory networks, mammalian behavioral dynamics, and others.^196^ Van der Meer *et al.* formulated an entropy estimator using the ratios of the forward and backward probability distributions of two consecutive transitions to quantify irreversibility.^153^ The authors also determined criteria whether the EPR estimator recovers total EPR or just a lower bound and this estimator also works with network topology with hidden cycles.^153,154^ In another study, Van der Meer *et al.* developed a framework to lower bound EP from measuring the time-resolved statistics of events.^197^

### 5.2 EPR calculation from fluctuation of currents

If the amount of heat delivered to the reservoirs or the gradients of forces applied on a nonequilibrium system is unknown, EP can be obtained from fluctuations of an observable, which is known as the variational characterization of the entropy production rate. Fluctuation–dissipation relation connects observable fluctuations and dissipation for physical systems operating at thermodynamic equilibrium. A new class of inequalities emerged that state dissipation constraints current fluctuations in steady states arbitrarily far from equilibrium, and is known as TUR.^198^ These relations capture the trade-off between the precision of a process and its thermodynamic cost. We will briefly discuss extensions and applications of the TUR, along with related results such as the kinetic uncertainty relation (KUR).

It has been shown that at the steady state, the dispersion or uncertainty of the time-integrated quantities like number of reactants and products or the number of steps taken by molecular motors are constrained by the thermodynamic cost associated with the process.^32^ If the thermodynamic cost of a chemical process consuming a total number of reactants *X* during a time interval *t*, the product of squared relative uncertainty and the total dissipation *Σ* is constant:^32,199,200^42
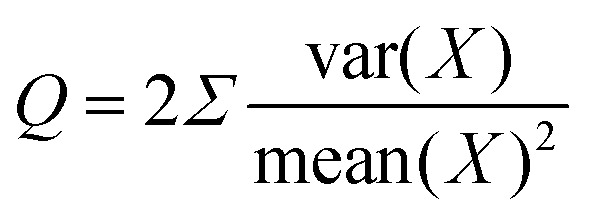
where *Q* is a dimensionless quantity, and var(*X*) and mean(*X*) represent the variance and mean of the variable *X*.

In 2016, Gingrich *et al.*^33^ derived a linear-response like bound on the large deviation of the current for Markov jump process and for small fluctuation limit uncertainty bound on the current fluctuation is shown. The authors also found nonequilibrium current fluctuations are more likely compared to the one obtained from linear-response relation for nonequilibrium systems. In 2018, Katarzyna *et al.* also used linear response theorem to obtain the relative uncertainty of the time integrated quantities, and their approach is applicable for systems with broken time-reversal symmetry.^201^ Andreas *et al.* rederived the thermodynamic uncertainty relation in 2020.^202^ Gianmaria *et al.* derived a generalization of the so-called TUR without needing the large deviation theory or information-theoretic techniques.^34^ In 2016, Patrick *et al.* derived a universal parabolic bound on the generating function of an arbitrary current which depends solely on the average entropy production.^203^ They also obtained a power-efficiency trade-off of heat engines working under the temperature gradient between two heat baths. For broken time reversal symmetry, the bounds on the relative uncertainty are controlled both by dissipation and by a parameter encoding the asymmetry of the Onsager matrix.^204^

TUR has been extended to finite-times,^205^ discrete times,^206^ multidimensions,^35^ systems following generalized Langevin equation with memory,^38^ underdamped Langevin dynamics,^38^ unidirectional processes,^207^ optimal TUR for Markov process,^208^ TUR including measurement and feedback,^209^ and for arbitrary initial states.^210^ A recently derived thermodynamic uncertainty relation shows that the minimum scaled variance of a charge, as well as the charge's variance, is governed not only by the mean entropy production but also by the higher moments of its probability distribution.^200^

Ivan *et al.* derived a new inequality called kinetic uncertainty relation (KUR) that states how observable fluctuations in discrete stochastic systems are bounded by the mean number of jumps among discrete states of stochastic systems, applicable for all times.^130^ There have been unified thermodynamic and kinetic uncertainty relation.^211^ A tighter bound on the current fluctuation is achieved from both thermodynamic and kinetic uncertainty relation, and it leads to a stronger classical speed limit. This framework can also be extended to first passage time observables and is applicable for systems with unidirectional transitions.^212^

Gingrich *et al.* derived an inequality relating the dissipation rate to current fluctuations in jump processes and found a lower bound on the total dissipation rate for driven diffusive process from observed coarse-grained currents.^213^ Li *et al.* estimated entropy production rate from probability currents.^214^ Using the finite-time generalization of the thermodynamic uncertainty relation, the mean and fluctuations of the entropy production were obtained, and the framework requires short experimental time-series data.^215^ It was further used to infer a lower bound to entropy production rate from flickering data generated by interference reflection microscopy of HeLa cells.^216^

So far, we have discussed variants of the TUR based on transition currents. TUR, however, has also been extended to account for fluctuations in first-passage times.^217,218^ In this formulation, the TUR is expressed as43
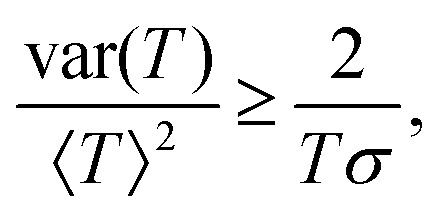
where 〈*T*〉 and var(*T*) denote the mean and variance of the first-passage time, respectively, and *σ* is the entropy production rate. More recently, the TUR framework has been extended to bound the characteristic timescale 
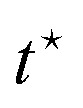
 over which a system can exhibit anomalous diffusion, where the mean-squared displacement follows a power law in time, 〈*x*^2^(*t*)〉 ≃ *D*_*α*_*t*^*α*^, with generalized diffusion coefficient *D*_*α*_ (units: m^2^ s^−*α*^) and anomalous exponent *α* ≠ 1:^219^44
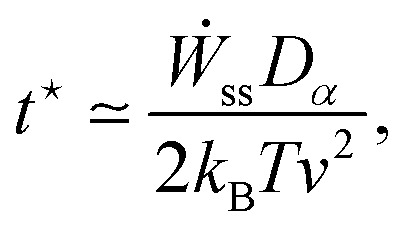
where *Ẇ*_ss_ is the steady-state dissipation rate, and *v* is the mean particle velocity, defined through the linear growth of displacement, 〈*x*(*t*)〉 = *vt*.

### 5.3 EPR from optimization

For discrete-time models, Ehrich^156^ introduced *σ*_fit_, which searches over possible hidden dynamics consistent with the observed statistics, given knowledge of the number of hidden states. The optimization problem was formulated for a four-state system, with two states observed and the other two coarse-grained into one. This approach can be extended to similar settings, provided that the number of observed states matches the number of hidden states and the jump probability matrices are invertible.

In continuous-time models, Skinner and Dunkel^220^ derived a lower bound on the total EPR, *σ*_2_, by minimizing it over a canonical representation of the system that preserves both first- and second-order transition statistics. The canonical form is obtained through a sequence of transformations that do not increase the EPR while maintaining the mass rate statistics up to second order. They further showed that for any triplet of coarse-grained states *I*, *J*, *K*, the number of intermediate states within *J* connected to *I* and *K* can be reduced to at most four without affecting the minimum EPR. Because the canonical form has a simple structure, one can readily identify the constraints ensuring that the observed statistics are preserved.

In another study, Skinner and Dunkel^196^ proposed an optimization method to estimate the EPR (*σ*_T_) in systems with two observed states using waiting-time statistics. By rescaling the rates and steady-state probabilities of the underlying system, they obtained an estimator expressed as a factor 
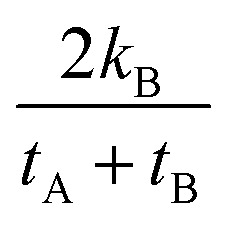
 multiplied by a function *Λ*, which depends solely on the ratio 
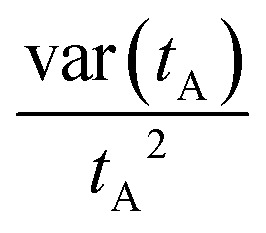
, where *t*_A_ and *t*_B_ are the waiting times in the observed states. The function *Λ* can be computed numerically by solving an optimization problem for each value of 
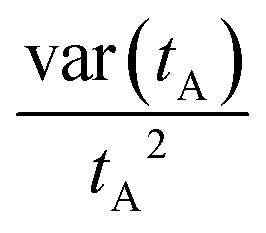
.

**Fig. 6:**
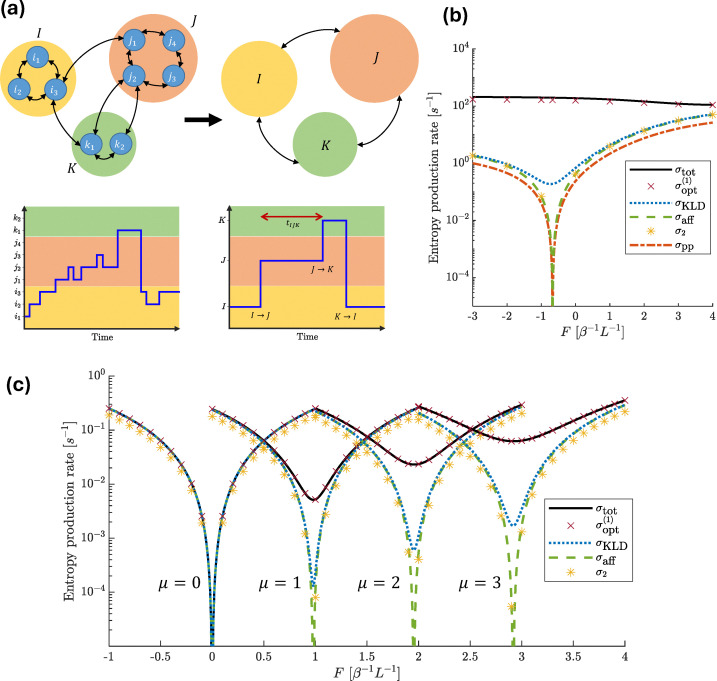
(a) The full Markovian system consists of the substates (*i*_*n*_, *j*_*n*_, and *k*_*n*_) (left), while the coarse-grained system consists of the mesostates (*I*, *J*, and *K*) (right). An example of a full trajectory (left), illustrating transitions between substates, and the corresponding coarse-grained trajectory (right), showing transitions only among the observed states. (b) Comparison of different EPR estimates for the four-state model shown in Fig. 3(a): the total EPR, *σ*_tot_ (solid black line); our bound, *σ*^opt^_1_ (brown cross), calculated using eqn (46); the KLD estimator, *σ*_KLD_ (dotted blue line); the affinity estimator, *σ*_aff_ (dashed green line); the two-step estimator, *σ*_2_ (yellow asterisk); and the passive partial entropy production, *σ*_pp_ (dash-dotted orange line). (c) EPR estimates for the molecular motor model shown in Fig. 3(b): the total EPR, (*σ*_tot_) (solid black line); our bound, (*σ*^opt^_1_) (brown cross); the KLD estimator, (*σ*_KLD_) (dotted blue line); the affinity estimator, *σ*_aff_ (dashed green line); and the two-step estimator, *σ*_2_ (yellow asterisk). The details of the parameters used can be found in ref. 150. This figure has been reproduced from ref. 150 with permission from the American Physical Society, copyright 2026.

In another study,^150^ we introduced a new method that incorporates additional information about the underlying system topology, which was not accounted for in earlier approaches and found EPR for a partially observed system using an optimization techniques based on the statistics of the transition rates and the cumulants of the waiting times (Fig. 6). Given a coarse-grained system and a model of the full Markovian network topology, we formulated an optimization problem to obtain a tight bound on the total EPR. The observables considered include the coarse-grained steady-state probabilities, π_*I*_, representing the probability of being in state *I*; the first-order mass rates (steady state probability density times the transition rates), *n*_*IJ*_, corresponding to transitions *I* → *J* (Fig. 6(a)); the second-order mass rates, *n*_*IJK*_, associated with transitions *I* → *J* → *K*, and the conditional waiting-time distributions *Ψ*_*IJK*_(*t*), describing the distribution of waiting times in state *J* before transitioning to *K*, conditioned on a prior transition *I* → *J*. The optimization problem was defined over all possible underlying systems with the same topology as the assumed Markovian model that reproduce the observed statistics, while minimizing the EPR. By construction, the EPR of the coarse-grained system is bounded from below by the minimal entropy production of the underlying Markovian system consistent with the observed statistics.

We first derived analytical results for the Laplace transforms of conditional probabilities of two- and three-state transitions, which enabled us to formulate the optimization problem.^150^ Let *S* denote the true underlying Markovian system and *R* a general system with the same topology as *S*, that is, the same states and allowed transitions, but arbitrary mass rates and steady-state probabilities. Considering all systems *R* with steady-state probabilities π^*R*^_*I*_ = π^*S*^_*I*_, first-order mass rates *n*^*R*^_*IJ*_ = *n*^*S*^_*IJ*_, second-order mass rates *n*^*R*^_*IJK*_ = *n*^*S*^_*IJK*_, and conditional waiting-time distributions *Ψ*^*R*^_*IJK*_(*t*) = *Ψ*^*S*^_*IJK*_(*t*), the following inequality holds between the EPR of *S* and *R*:45
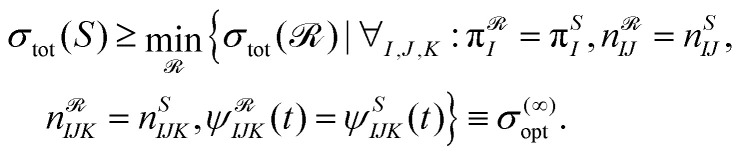


Unlike constraints on steady-state probabilities and mass rates, enforcing equality of the full waiting-time distributions *Ψ*_*IJK*_(*t*) requires matching continuous functions, which cannot be exactly reconstructed from finite trajectory data. Moreover, directly imposing functional constraints with non-trivial dependence on optimization variables is computationally demanding. To address this, we reformulated the optimization problem using the moments of the waiting-time distributions:46
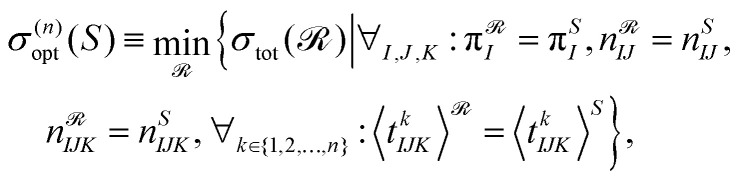
where 〈*t*^*k*^_*IJK*_〉 denotes the *k*-th moment of *Ψ*_*IJK*_(*t*).

The analytical expressions for the moments can be obtained from the Laplace transform as 
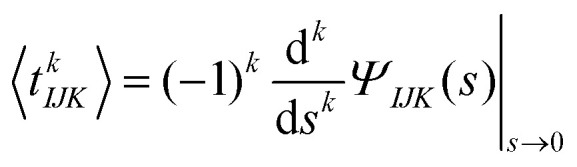
. This reformulation simplifies the dependence of the observables on the optimization variables, making the problem more tractable. After evaluating the observables, the optimization is solved using a global non-linear search algorithm.^221^

Fig. 6 shows different EPR bounds on a four-state system (Fig. 6(b)) and a molecular motor model (Fig. 6(c)).^150^Fig. 6(b) shows comparison of existing estimators like *σ*_2_, *σ*_KLD_ (*Ṡ*_KLD_ in eqn (38)), *σ*_aff_ (*Ṡ*_aff_ in eqn (39)), and *σ*_pp_ (eqn (24)) with our estimator *σ*^(1)^_opt_ (eqn (46) with *n* = 1). We conclude that, at the stalling force (condition for no current in the visible link) we obtain *σ*_pp_ = *σ*_aff_ = *σ*_2_ = 0, which corresponds to the trivial bound. In contrast, *σ*_KLD_ and our estimator *σ*^(1)^_opt_ provide non-trivial bounds. Moreover, *σ*^(1)^_opt_ significantly surpasses *σ*_KLD_ and yields a tight bound. For this 4 state system (Fig. 3(a)), using higher moments to calculate *σ*^(2)^_opt_ (eqn (46) with *n* = 2) did not lead to any improvement compared to *σ*^(1)^_opt_. While *σ*_2_ can be larger than *σ*_KLD_ in some cases,^220^ for the rate values considered here, we find *σ*_2_ < *σ*_KLD_. In fact, although *σ*_2_ and *σ*_aff_ appear to be similar in Fig. 6(a), we confirmed that *σ*_2_ < *σ*_aff_ for all values of *F* used in the transition rates. We found the following hierarchy of the different EPR estimators for the molecular motor given the transition rates considered: *σ*^(1)^_opt_ ≥ *σ*_KLD_ ≥ *σ*_aff_ ≥ *σ*_2_. At the stalling force for each value of *μ*, we found *σ*_aff_ = *σ*_2_ = 0, corresponding to the trivial bound. In contrast, similar to the four-state system. In Fig. 6(b), *σ*^(1)^_opt_ significantly surpasses *σ*_KLD_ and provides a tight bound.

Other EPR estimators that use observed transitions only. For example, Dominic *et al.*^220^ introduced EPR estimators from observed transition statistics using an optimization framework, which would produce non-vanishing values for processes that appear to maintain detailed balance and time-symmetric. They used their framework for bacterial flagella motors, calcium oscillations within human embryonic kidney cells, and growing microtubules.

### 5.4 EPR from thermodynamic speed limit

EPR has also been estimated from speed limits.^66,222–224^ The fastest rate (*τ*_*ζ*_^−1^) at which any physical observable can measurably change is limited by the rate (*τ*^−1^) at which the probability distribution can change.^225^ The thermodynamic speed limit can be mathematically expressed as

47 *τ*^−1^ ≥
*τ*_*ζ*_^−1^.

The intrinsic speed *τ*^−1^ is related to the time parameterized Fisher information^226^ (*I*_F_(*t*)) *via*
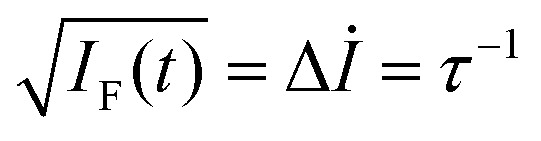
. The thermodynamic speed (*τ*_*ζ*_^−1^), defined by the ratio of the function's rate of change to one standard deviation of the associated variable, 
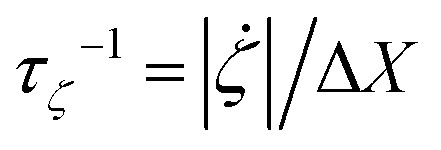
, where 
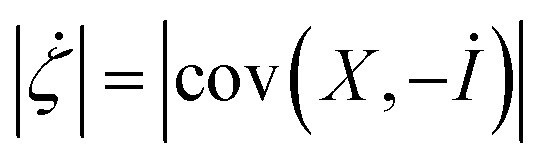
 is the covariance between the surprisal rate and observable or variable *X*.

In another study,^227^ Nicholson *et al.* proposed a theoretical framework to derive the thermodynamic speed of observables (*X*) in models where the full probability distribution is known, based on linear regression of the surprisal rate (*İ* = −*d*_*t*_ ln *P*(*X*,*t*)):

48 *İ* = *bX* +
*a*.

They showed that the optimal intercepts (*a*_opt_) and optimal slopes (*b*_opt_) of the linear regression model are related to free energies and observable speed, respectively.^227^ Consider the rate of change of (log) probability, *r̂* to be a function of the driven variables, *X*, where *X*^T^ = (*X*_1_,*X*_2_,…,*X*_*N*_). For the function

49 *r̂* =
*a* + *b*^T^*X* + ℰ,

we can find the optimal parameters by minimizing the mean-squared error:

50 〈ℰ^2^〉 = 〈(*r̂* − (*a* +
*X*^T^*b*))^T^(*r̂* −
(*a* +
*X*^T^*b*))〉.

Setting 
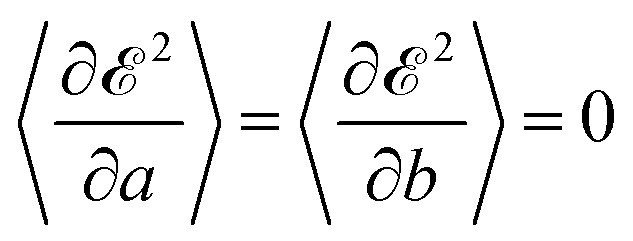
, gives

51 *a* = 〈*r̂*〉 −
〈*X*^T^〉*b*

and

52 〈*XX*^T^〉*b* =
〈*X*(*r̂* − *a*)〉.

Solving eqn (52), we find the optimal slope53
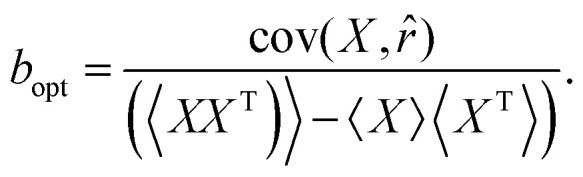


The covariance in the numerator corresponds to physical observables (*e.g.*, heat rate, rate of dissipated work, entropy production rate). Substituting *b*_opt_ into eqn (51), we find the optimal intercept, *a*_opt_. For example, if the observable is the system's energy, the observable speed corresponds to the heat dissipation rate (assuming no work is performed in the process). However, evaluating the time-parameterized Fisher information still requires access to the full probability distribution. The theory was then applied to discrete state models.^227^

We recently showed that the need for the full probability distribution can be bypassed by considering the coordinate transformation of the Fisher information which would relate the coordinate-transformed Fisher information to the variance of an experimental variable.^195^ This method enables us to use the statistical moments of the experimental observables to obtain the intrinsic speed. This coordinate-transformed Fisher information is further used to calculate the dissipation rate using the variance of observable responsible for dissipation using:54
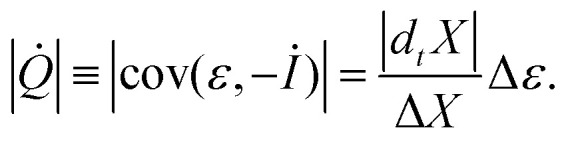


**Fig. 7:**
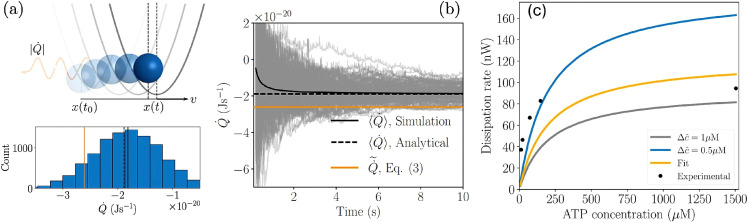
(a) A Brownian particle releases energy in the form of heat when it is dragged through a viscous medium. At time *t* = *t*_0_, the particle is located at the center of the trapping potential, *x*(*t*_0_). As the trap is translated, the particle's average position shifts upward along the potential landscape. Throughout this process, heat is dissipated into the surroundings at a rate (|*Q̇*|). A histogram of the heat transferred to the environment, obtained from simulations of overdamped Langevin dynamics with 10 000 noise realizations at *t* = 4 s is shown. The housekeeping heat rate predicted by eqn (55) (eqn (3) in the legend) (orange) matches both the sample mean from the simulations (black solid line) and the analytical mean (black dotted line). (c) Comparison of the heat rates predicted using eqn (56) for different ATP concentration uncertainties (gray and blue solid lines), experimentally measured average dissipation rates (shown by black filled circles), and a fit based on a chemical kinetics model is shown as a function of ATP concentration for a microtubule active gel. This figure has been reproduced from ref. 195 with permission from the American Physical Society, copyright 2026.

*X* could be the position of a particle exhibiting a nonequilibrium process, concentration, number of molecules of a chemical species, *etc.* The key advantage of our method is to experimentally exploit the known thermodynamic speed limit to calculate the dissipation rate. This method is not only limited to entropy production estimation, moreover, *ζ* can represent different thermodynamic variables depending on the observable *X*. We illustrated our theoretical approach to estimate dissipation rates for two systems: (1) harmonically bound Brownian particle dragged by a constant velocity (Fig. 7(a) and (b)) and (2) active gel composed of microtubule and kinesin molecule that are driven out of equilibrium by ATP hydrolysis (Fig. 7(c)).

For harmonically dragged Brownian particle, we predicted the heat dissipated using only the measured uncertainty in position Δ*x*, trap speed *v*, and the temperature of the surrounding fluid *T*:55
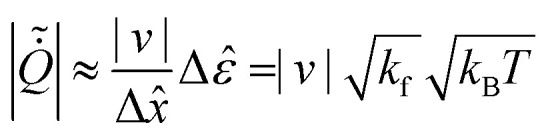


The first expression of the heat rate can use data directly. We estimated 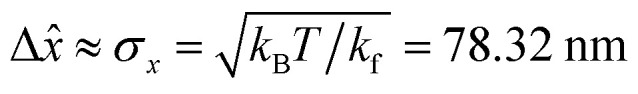 (errors can be of order ±2 nm) and Δ*ε̂* ≈ *k*_B_*T*/2. In this case, our prediction used quantities that are experimentally controlled or can be experimentally measured. As validation of the numerical prediction, we used the dissipation rate by numerically simulating Langevin dynamics. Our predicted value 
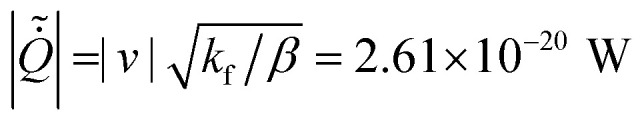
 agrees well with the value from our numerical simulations 1.85 × 10^−20^ W. Fig. 7(b) shows our prediction also agreed well with an analytical formula for the heat rate |〈*Q̇*〉| = *v*^2^/*γ* = 1.885 × 10^−20^ W.^228^

For the active gel system, the predicted heat rate from our formalism based on coordinate-transformed thermodynamic speed (Fig. 7(c)) limit^195^56
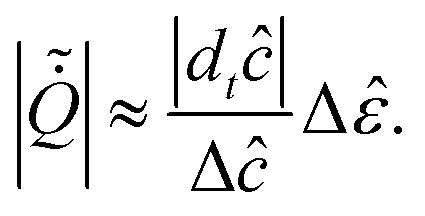


In this case, we did use a chemical kinetics model to estimate the dissipation rate because the time derivative of concentration, *d*_*t*_*ĉ*, has not yet been reported. However, when available, *d*_*t*_*ĉ* can be directly used to estimate the heat rate. As part of the expanded discussion, we describe in more detail how we estimated the energy fluctuations to be 10^−8^ J or 10 nW in the active gel. We first estimated this value based on Foster *et al.*'s experimental measurements of the heat release rate.^45^ Over 7 minute intervals for a total of 180 minutes, their measurements show fluctuations on the order of 10–15 nW when the gel was prepared with and without the pyruvate kinase-based ATP regeneration system. As a point of comparison, these fluctuations are smaller than the mean heat release rate they measured: the microtubule active gel dissipates energy at a rate on the order of 100 nW. The experimental uncertainty is 0.2 nW energy resolution of the picocalorimeter.^45^ Since 10 nW is an order of magnitude smaller than the measured heat release and two orders of magnitude larger than the experimental error, we used the 10 nW deviations as an estimate of the energy fluctuations.

Our formalism can also be extended into higher dimensions. One of such example is shown here. Suppose we consider a moving trap along the *x*-coordinate, while the particle remains equilibrated and static in the *y*-coordinate. If the *y*-coordinate is equilibrated and does not contribute to dissipation, then using *y* alone to estimate the heat rate would yield a poor estimate unless it is coupled to the driven *x*-coordinate.

When the equilibrated *y*-coordinate is coupled to the driven *x*-coordinate, the covariance cov(*x*,*y*) captures this coupling. In this two-dimensional case, one can perform a linear regression for the surprisal rate, *r̂*(*x*,*y*), of the form

57 *r̂* = *ax*
+ *by* + *c*.

The variance of *r̂*,

58 *τ̃*^−2^ ≡ *Ĩ*_F_ ≡
Δ*r̂*^2^,

represents the coordinate-transformed Fisher information. Expressed in terms of position uncertainties, this Fisher information becomes59
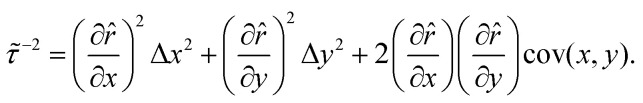


In this scenario, if the driven *x*-coordinate is inaccessible, the terms involving (∂*r̂*/∂*x*) vanish. The optimal parameters for the linear statistical model then give (∂*r̂*/∂*y*) = ∂_*t*_*y*/*σ*_*y*_^2^. Since *y* is equilibrated, ∂_*t*_*y* vanishes, leading to a poor estimate of the heat rate when only the *y*-coordinate is used in the coordinate transformation.

### 5.5 EPR from machine learning approaches

Kim *et al.*^229^ proposed a neural estimator for entropy production (NEEP), that estimates EP from trajectories of relevant variables without detailed knowledge of system dynamics, applied their approach to bead spring and discrete flashing ratchet models. Their method can estimate coarse-grained EP even for higher-dimensional data. This approach is independent of the system dynamics, and can be useful for other contexts.^230^

Kim *et al.*^231^ also introduced a machine-learning framework to estimate EP in systems with odd-parity variables using neural networks trained on trajectory data and parity information. They further demonstrated their approach with an underdamped bead-spring model and an odd-parity Markov jump process. Odd-parity systems require additional estimators to account for asymmetry (Δ*S*_as_) and waiting-time contributions (Δ*S*_WTD_). The bead-spring model and the Markov jump process used two and three such estimators, respectively. Related studies^99,220^ found out that WTD fluctuations can reveal EP in hidden Markov or semi-Markov processes.

We also studied the RNEEP estimator, *σ*_RNEEP,*m*_, which approaches the entropy production rate as an optimization task solved by stochastic gradient descent.^229,232,233^ Its input is a collection of sequences of length *m* taken from a long trajectory, and the output is the coarse-grained entropy production per step. Similar to the Plug-in estimator mentioned in Section 5.1, RNEEP relies only on discrete state sequences and does not require explicit knowledge of waiting-time distributions. A recurrent neural network can be used to compare forward and time-reversed sequences, thereby quantifying trajectory irreversibility. Although its estimates are typically close to those from the plug-in or affinity-based approaches, the RNEEP framework offers a flexible, machine-learning-based method for refining lower bounds on entropy production. The EP estimates from RNEEP are further discussed in Section 5.7. Moreover, there are other studies where machine learning approaches have been used to estimate dissipation.^234^

### 5.6 EPR from error propagation

Di Terlizzi *et al.* introduced a variance sum rule (VSR) for displacement and force variances that allows direct EP estimation in steady.^235,236^ They demonstrated their approach to estimate EP for active Brownian particles in optical traps and red blood cell flickering that agree with calorimetric measurements. The authors further generalized the VSR^235^ relating variances of displacement and force for overdamped Langevin systems at NESS. They found out deviation of mean-squared displacement from normal diffusion is a result of non-equilibrium condition. The VSR highlights that deviations from normal diffusion are caused by nonequilibrium effects. They derived entropy production rate which depends on second-order time derivatives of position correlations, and applied their framework to exactly solvable models. This approach can infer the hidden nonequilibrium behavior.^235^ Our method^195^ as presented in Section 5.4 is a generalized version of VSR to estimate EP.

### 5.7 EPR comparison

This section compares inferred dissipation bounds from spatiotemporally coarse-grained data. Passive partial EP *σ*_pp_ (eqn (24)) provides a lower bound for the total entropy production. The total entropy production is given by60
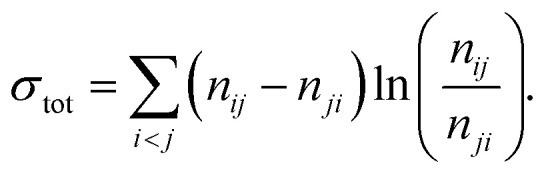


Restricting the sum to a pair of observed states *i* and *j* yields61
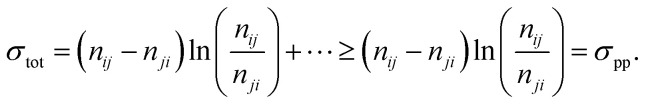


Ref. 129 showed

62 *σ*_tot_ ≥ *σ*_ip_ ≥
*σ*_pp_ ≥ 0,

where using a molecular motor model system, Martínez *et al.* showed^99^

63 *σ*_KLD_ ≥ *σ*_aff_
≥ *σ*_pp_.

Mean EPR rates can be estimated using the following formula for different CG methods^78,130,131^64
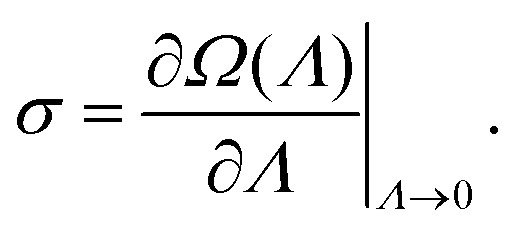
where *Ω* represents the dominant eigenvalue of a coarse-grained or tilted transition rate matrix as expressed below65
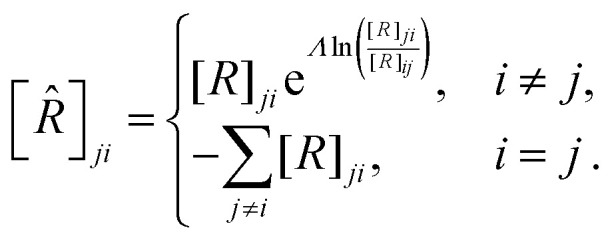
where [*R̂*]_*ji*_ would vary depending on the coarse-graining method employed, and different forms of the matrix are summarized in ref. 128. We investigated various notions of partial information in driven systems and the corresponding mean EPRs.^128^ TM refers to total mean EPR considering full knowledge about system dynamics.

We discussed mainly two approaches. One is lumping (Fig. 5(a)) and the other one is decimation (Fig. 5(d)). We call EPRs obtained from different CG methods as SCGF, AM, L, and compare them with partial information-based inference like IPEP as discussed in Section 4.2. SCGF refers to a decimation-based CG approach which employs scaled cumulant generating function (SCGF). Whereas, AM refers to a decimated or coarse-grained system dynamics that follows an approximated Master equation with effective transition rates. L refers to a lumping method^74^ where the probabilities of the merged states are summed up, while the steady states probabilities of the rest of the states remain unchanged, and the coarse-grained system dynamics follow an approximated Master equation. Another lumping procedure (HS) was developed by Hummer and Szabo.^160^ Their method ensures that the time-dependent occupancy number correlation functions in the coarse-grained system are equal to the ones of the original system. The same reduced matrix (rate matrix for the coarse-grained system) can be obtained from the projection operator technique. In the approximated Markovian limit, the reduced transition rate matrix was calculated analytically.^160^

**Fig. 8:**
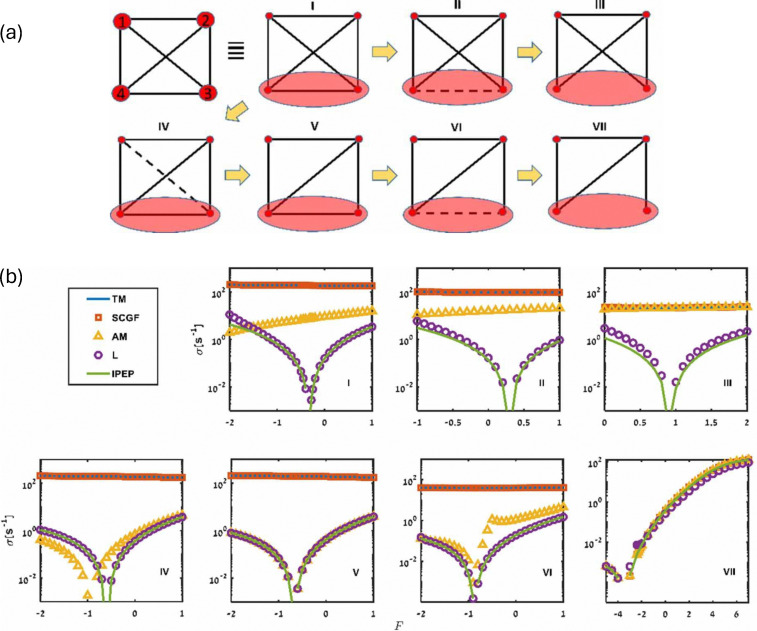
Different network topologies in which states 1 and 2 are observed, while states 3 and 4 are indistinguishable, are shown. States 3 and 4 are shaded in red and are collectively referred to as the “hidden state.” The weights of the 3–4 and 1–3 links are varied, resulting in the different network topologies shown. (b) The total mean (TM) is calculated using a fully resolved network, and the coarse-grained mean EPRs estimated using eqn (65) are compared for each topology. The EPR from the scaled cumulant generating function (SCGF) is obtained after decimating states and applying a Markovian approximation. The EPR obtained from a coarse-grained network by applying lumping – while preserving transition fluxes and steady-state probabilities during the coarse-graining process – is denoted by L. The EPR obtained from the approximated master equation is called AM, and informed partial entropy production (IPEP) is based on transition rates among the observed states and the stalling probability distribution. This figure has been reproduced from ref. 128 with permission from the IOP Publishing, copyright 2026.

Fig. 8(a) shows the different network topologies used to compare EPR estimators in Fig. 8(b), in which TM, SCGF, AM, L, and IPEP correspond to *σ*^TM^, *σ*^SCGF^, *σ*^AM^, *σ*^L^, and *σ*^IPEP^, respectively. TM provides the mean EPR for fully observed systems, so the closer the EPR bounds are to TM, the better the estimators. Coarse-graining *via* scaled cumulant generating functions (SCGF), which involves decimation and redistribution of steady-state probabilities and transition rates, accurately reproduces the total mean EPR as expected. SCGF approach was meant to preserve the total mean EPR (*σ*^TM^ or TM).^78^ We compared AM, L, HS, and IPEP with the total mean EPR (TM) across all network topologies shown in Fig. 8(b). The approximated Master equation (AM) yields a lower bound on TM because decimation produces a non-Markovian coarse-grained system, preventing AM from capturing the full entropy production.

When the hidden substates are disconnected (network topology III and VII in Fig. 8(a)), AM provides values closer to the total mean EPR (*σ*^TM^) as no cycles carrying transition currents are lost during the coarse-graining method. For topology VII of Fig. 8(a), the available observations are sufficient to determine the total entropy production exactly, because the observed cycle is the sole entropy-producing fundamental cycle in the network; consequently, AM and IPEP equals the total EPR (TM). For specific topologies, reducing connectivity among hidden states leads the AM method to converge to the TM EPR, since the entropy contribution from loss cycles with transition flux diminishes.

By comparing state-based coarse-graining methods (lumping L and HS, and decimation-based SCGF and AM) that rely on knowledge of the network topology, with the mean EPR inferred solely from observed states and transitions (IPEP) in Fig. 8(b), we showed that the impact of partial information on inferred EPR depends strongly on network structure. Notably, IPEP yields entropic information comparable to lumping (L), which preserves steady-state properties, but their difference increases when hidden substates are disconnected, reflecting loss of hidden state information. The discrepancy between HS and L varies with stalling force and timescale differences: HS is sensitive to timescale separation between merged states, while L is not. These findings highlighted the interplay between topology, hidden connectivity, and timescale separation in entropy production inference. The framework can be extended to more complex systems with multiple hidden microstates or hidden cycle currents.

**Fig. 9:**
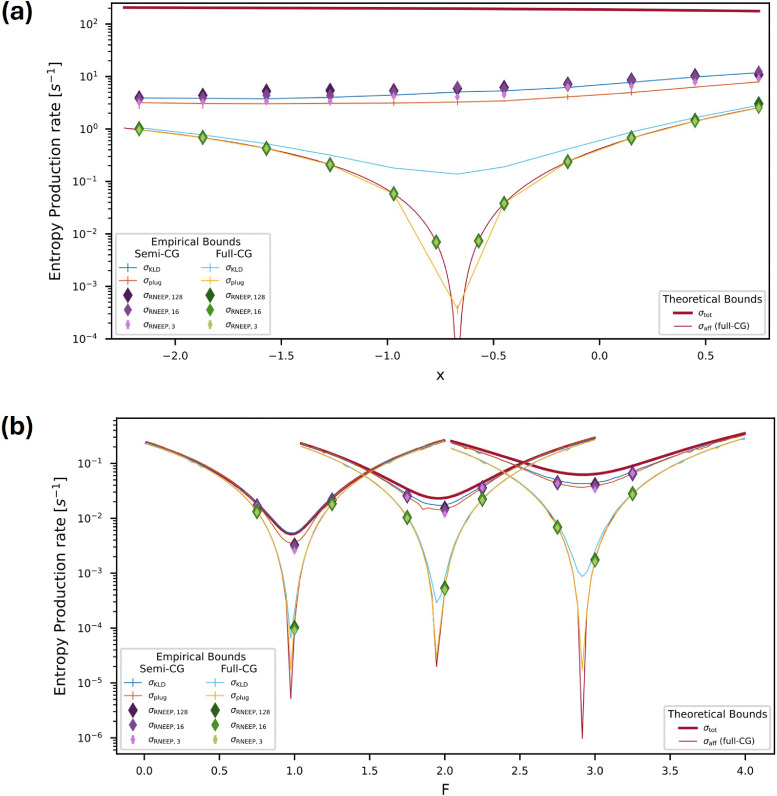
(a) Entropy production rates for the 4-state system. Total EPR, *σ*_tot_ (dark red); KLD estimator, *σ*_KLD_, for semi-CG (dark blue) and full-CG (light blue) data; plug-in estimator, *σ*_plug_, for semi-CG (dark orange) and full-CG (light orange) data; RNEEP estimator, *σ*_RNEEP,*m*_, for semi-CG (light to dark purple with increasing sequence length *m*) and for full-CG data (light to dark green with increasing sequence length *m*); and the affinity contribution, *σ*_aff_, for full-CG data (red). (b) Entropy production rates for the molecular motor model: the total EPR, *σ*_tot_ (dark red); the KLD estimator, *σ*_KLD_ (dark blue); the plug-in estimator, *σ*_plug_ (dark orange); and the RNEEP estimator, *σ*_RNEEP,*m*_ (light to dark purple for increasing sequence length (*m*)). This figure has been reproduced from ref. 152 with permission from the American Physical Society, copyright 2026.

In another work, we compared three estimators, the plug-in estimator (*σ*_plug_ (eqn (34) in Section 5.1), RNEEP estimator, and KLD estimator for different model systems described below. First, we discuss 4-state system, with three observed states among which 2 states are Markovian Fig. 4. In the full-CG case, the KLD estimator *σ*_KLD_ provides the tightest lower bound on the entropy production rate, while *σ*_RNEEP,*m*_ converges to the affinity *σ*_aff_ with increasing sequence length, and the plug-in estimator *σ*_plug_ shows bias near the stall force. For the semi-CG data, *σ*_RNEEP,*m*_ becomes comparable to *σ*_KLD_ for *m* ≈ 20, both outperforming *σ*_plug_. Thus, KLD dominates in the full-CG setting, whereas in the semi-CG setting, both KLD and RNEEP provide similarly tight bounds given sufficient sequence length. The results have been reproduced with permission and presented in Fig. 9(a).

Next, we discuss EP values comparison from three EPR estimators (*σ*_KLD_, *σ*_RNEEP,*m*_, *σ*_plug_) for the molecular motor system (Section 4) and analyzed in Fig. 9(b) under both the full-CG and semi-CG schemes for three values of Δ*μ* and range of *F* values including the stall force. As we can infer from Fig. 9(b), the semi-CG scheme consistently provides tighter bounds on the entropy production rate (EPR) than the full-CG case, since it exploits additional information from intra-transitions within macrostates. In the full-CG trajectories, the KLD, plug-in, and RNEEP estimators yield similar results, except near the stalling force, where *σ*_KLD_ remains the tightest bound. Under the semi-CG scheme, however, the transformed KLD estimator outperforms both the plug-in and RNEEP, producing the sharpest lower bound on EPR. The RNEEP estimator still improves with sequence length *m*, converging toward the KLD results, while the plug-in estimator eventually falls within the error of *σ*_RNEEP,*m*_. This difference between the semi-CG and full-CG frameworks highlights the role of hidden transitions in encoding irreversibility, especially evident near the stalling force.

## 6 Conclusions

Substantial progress over the past two decades has greatly advanced our understanding of thermodynamic irreversibility in systems operating far from equilibrium, clarifying how dissipation, entropy production, and broken time-reversal symmetry are connected.^237^ Foundational questions have been explored, including the relationship between information-theoretic entropy production (ITEP) and thermodynamic EPR, how irreversibility can be estimated from partial observations, how coarse-graining modifies entropy-production estimates, and how the parity of coarse-grained observables under time reversal affects the definition of broken time-reversal symmetry. Optimization principles—such as minimal entropy production and optimal transport—have provided unifying structure, and significant effort has been devoted to identifying measurable signatures of non-equilibrium behavior.

Despite this progress, determining whether a system operates at equilibrium or out of equilibrium remains difficult in many experimental contexts. In several scenarios – including one-dimensional driven variables with non-Markovian statistics,^238^ Gaussian observables, or multidimensional linear Markovian systems with multiple timescales^239^ – equilibrium *versus* nonequilibrium behavior can only be distinguished through invasive perturbations. This highlights an urgent need for reliable, noninvasive approaches to detect time irreversibility directly from experimental time-series data.

Although entropy production and broken time-reversal symmetry remain the primary markers of irreversibility, other indicators such as effective temperature^240^ are often invoked. Recent experiments on active fluctuations of an AFM cantilever tip embedded in the mitotic cell cortex reveal a striking decoupling between effective temperature and entropy-production rate,^241^ underscoring the limitations of effective temperature as a universal metric. Additional measures, such as response functions and transfer entropy, provide complementary perspectives on temporal asymmetry.^242^

As stochastic thermodynamics interfaces with fields such as active matter, soft materials, machine learning, and field theory, new classes of questions have emerged that go beyond traditional thermodynamic settings. In active matter systems,^243–245^ quantifying dissipation in the presence of persistent non-equilibrium fluxes remains a central challenge. In machine learning, thermodynamic inference is increasingly used to analyze learning dynamics, model complexity, and information bottlenecks. Meanwhile, field-theoretic frameworks are being developed to describe fluctuations, symmetry constraints, and coarse-grained entropy production in spatially extended systems. These cross-disciplinary links are reshaping conceptual foundations and prompting new questions about universality, inference, and scalability in complex driven systems.

Parallel theoretical and experimental advances, especially in single-molecule tracking and manipulation, have paved the way for engineering synthetic materials capable of performing functional tasks such as catalysis,^246^ directed motion,^247^ and autonomous propulsion.^248^ While stochastic thermodynamics was originally built on explicit knowledge of underlying system dynamics, recent model-free approaches^249^ aim to infer thermodynamic quantities directly from observed trajectories. Such methods promise to connect experiments more directly with theory, especially for synthetic living systems characterized by strongly coupled degrees of freedom, memory effects, and finite-time dynamics. Machine learning-based inference frameworks may play a prominent role in this direction.

Looking forward, major open challenges include establishing model-free theoretical frameworks with predictive power for multivariate driven systems, and exploiting biased-ensemble methods for reverse engineering active materials.^237^ Continued integration of theoretical, computational, and experimental perspectives will be essential for uncovering universal principles of non-equilibrium physics and enabling the design of next-generation synthetic systems.

## Author contributions

All authors contributed equally to this work.

## Conflicts of interest

There are no conflicts to declare.

## Data Availability

No primary research results, software or code have been included, and no new data were generated or analyzed as part of this review.
